# High‐Period Element Doping as a Key Driver of Hydrogen Evolution in a Proton Exchange Membrane Water Electrolyzer

**DOI:** 10.1002/smll.202507086

**Published:** 2025-08-08

**Authors:** Jae‐Hoon Baek, Se Jung Lee, Na Hyun Kim, Seung Min Lee, Jeong‐Min Seo, Hyuk‐Jun Noh, Jong‐Pil Jeon, Changqing Li, Sang Kyu Kwak, Do Hyung Kweon, In‐Yup Jeon, Jong‐Beom Baek

**Affiliations:** ^1^ Department of Energy and Chemical Engineering/Center for Dimension‐Controllable Organic Frameworks Ulsan National Institute of Science and Technology (UNIST) Ulsan 44919 Republic of Korea; ^2^ Department of Chemical and Biological Engineering Korea University 145 Anam‐ro, Seongbuk‐gu Seoul 02841 Republic of Korea; ^3^ Hydrogen Fuel Cell Research Center Korea Institute of Science and Technology (KIST) Seoul 02841 Republic of Korea; ^4^ Department of Chemical Engineering Wonkwang University 460, Iksandae‐ro Iksan Jeonbuk 54538 South Korea

**Keywords:** electrocatalyst, high‐period heteroatom doping, hydrogen evolution reaction (HER), metal–support interaction, proton exchange membrane water electrolysis (PEMWE)

## Abstract

Proton exchange membrane water electrolysis (PEMWE) is a promising strategy for sustainable hydrogen production, but its application is limited by the high cost and instability of catalysts under acidic operation conditions. Here, the study reports group VA element‐doped graphitic nanoplatelets (XGnPs; X = N, P, or Sb) as effective supports to enhance both the activity and durability of electrocatalysts. The resulting platinum (Pt) nanoparticles on XGnPs (Pt@XGnPs) catalysts exhibit improved charge transfer to the metal and strong metal–support interactions. Among them, Pt@SbGnP exhibits the best performance, with a low overpotential of 15.3 mV at 10 mA cm^−2^ and a Tafel slope of 27.8 mV dec^−1^, surpassing commercial Pt/C. System‐level testing further confirmed its superiority, achieving 68.2 mA cm^−2^ at 1.9 V with 96.6% Faradaic efficiency for two‐electrode system and 1 A cm^−2^ at 1.724 V for full PEMWE system. Density functional theory calculations reveal that heteroatom doping modulates the charge transfer to the metal, facilitating efficient hydrogen evolution reaction (HER) kinetics.

## Graphical abstract

Group VA element‐doped graphitic nanoplatelets (XGnPs, X = N, P, or Sb) enable strong electronic metal‐support interaction (EMSI) with platinum (Pt) nanoparticles, boosting hydrogen evolution reaction (HER) activity and durability. Among them, Pt nanoparticles on SbGnP (Pt@SbGnP) exhibit superior performance in both half‐cell and full proton exchange membrane water electrolysis (PEMWE), driven by enhanced charge transfer and surface area.

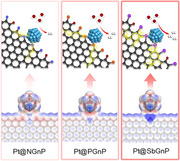

## 1 Introduction

As environmental pollution and climate change issues become more serious around the world, attention and knowledge are being applied to efforts to build a more sustainable and organized hydrogen economy.^[^ ^1^ ^]^ Water electrolysis for the production of hydrogen without pollution has attracted significant interest, and proton exchange membrane water electrolysis (PEMWE) in particular is considered one of the promising options.^[^ ^2^ ^]^ However, the widespread adoption of PEMWE is hindered by significant drawbacks, including its high cost and stability issue, which are largely attributed to the use of platinum (Pt)‐based catalysts. Various studies have been conducted to help catalysts withstand the harsh driving environments in acidic conditions, although commercialization is still hampered by high cost, insufficient efficiency, and limited stability.^[^ ^3^ ^]^ To overcome these limitations, extensive research is being conducted to reduce the use of platinum and improve stability.

The doping of heteroatoms onto carbon supports is widely regarded as an effective strategy for tailoring electronic structure and chemical properties, while preserving beneficial electrical conductivity and physicochemical stability.^[^ ^4^, ^5^ ^]^ It can also form activation regions, which enhance dispersion and prevent the aggregation of metal nanoparticles (NPs), ultimately improving catalytic activity and stability.^[^ ^6^, ^7^ ^]^ While extensive research has been conducted on heteroatom‐doped graphitic frameworks to develop efficient catalysts, most studies have focused on non‐metallic, low‐period elements such as boron (B), nitrogen (N), oxygen (O), phosphorus (P), and sulfur (S).^[^ ^8^, ^9^, ^10^, ^11^, ^12^, ^13^ ^]^ Despite these advances, the role of high‐period group VA elements in modulating metal–support interactions remains largely unexplored, representing a significant knowledge gap. Here, we investigate the effects of doping nitrogen (N), phosphorus (P), and antimony (Sb), a same‐group element, on carbon supports, and uncover their distinct influences on catalytic properties.^[^ ^14^, ^15^, ^16^ ^]^

To the best of our knowledge, this study is the first to systematically compare the effects of atomic periods on group VA dopants in Pt‐based catalytic systems. Among dopants, the Sb‐doped graphitic framework was expected to demonstrate the most superior performance for several key factors. On a same‐group element basis, as the period decreases, electronegativity increases, and in the opposite case, electropositivity decreases, which is a measure of an element's ability to donate electrons.^[^ ^17^, ^18^ ^]^ In this regard, Sb's high electropositivity facilitated substantial electron transfer to Pt NPs, which serve as the active sites for the hydrogen evolution reaction (HER), thereby enhancing the reduction process.^[^ ^19^ ^]^

Unlike conventional non‐metal dopants, Sb's unique position as a metalloid introduces distinct electronic characteristics and charge transfer capabilities, which have rarely been applied to electrocatalytic systems. Furthermore, Sb possesses unique electronic characteristics, which enable more efficient electron transport compared to other low‐period element dopants. These properties originate from its partially occupied p‐orbital and vacant d‐orbital, which result from its position as a high‐period metalloid element in the periodic table. This enhances the electron transport, fostered strong electronic metal‐support interaction (EMSI) with Pt NPs, further boosting catalytic activity and stability.^[^ ^20^ ^]^ Last, Sb possesses the largest atomic radius among the possible doping elements in group VA, which results in a carbon support with a larger surface area compared to N‐ or P‐doped supports.^[^ ^21^ ^]^ This increased surface area can uniformly disperse Pt NPs, enhancing catalytic activity and the durability of the catalyst by mitigating particle agglomeration over time.^[^ ^22^ ^]^ Integrating these atomic‐scale advantages into the catalyst framework enables a new design strategy that optimizes charge transfer, structural stability, and nanoparticle dispersion simultaneously.

Herein, we demonstrate atomic‐period‐dependent charge transfer between Pt NPs and heteroatom‐doped graphitic nanoplatelets (XGnPs; X = N, P, or Sb). The Pt NPs on XGnPs (Pt@XGnPs) catalysts were prepared by depositing Pt NPs onto edge‐selectively functionalized graphitic nanoplatelets (GnPs), doped with group VA elements (N, P, or Sb). In this study, we focused on the most widely studied group VA heteroatoms as dopants for carbon‐based supports and further extended our scope to include the metalloid element Sb, which lies at the boundary of nonmetals and metals, to explore the broader effect of periodic trends. Spectroscopic characterizations, Raman spectra, and theoretical calculations revealed that the XGnPs doped with the higher period element exhibited enhanced charge transfer to Pt NPs through Pt−X bonds. Notably, Pt@SbGnP showed exceptional HER performance, serving as experimental validation that high‐period doping can significantly enhance EMSI and overall catalytic behavior. The superior HER performance of Pt@SbGnP proved that enhancement of EMSI by high‐period elemental doping was a key factor. As a result, Pt@SbGnP exhibited outstanding HER performance with an overpotential of 15.3 mV (@10 mA cm^−2^) and a Tafel slope of 27.8 mV dec^−1^, outperforming commercial Pt/C. In overall water splitting, Pt@SbGnP delivered 68.2 mA cm^−2^ at 1.9 V with a high Faradaic efficiency of 96.6%. Its excellent activity was further validated in a full‐cell PEMWE system, achieving 1 A cm^−2^ at only 1.724 V. These results clearly demonstrate that high‐period heteroatom doping, particularly with Sb, can effectively boost EMSI and catalytic performance in both half‐cell and practical full‐cell systems.

## 2 Results and Discussion

**Figure 1:**
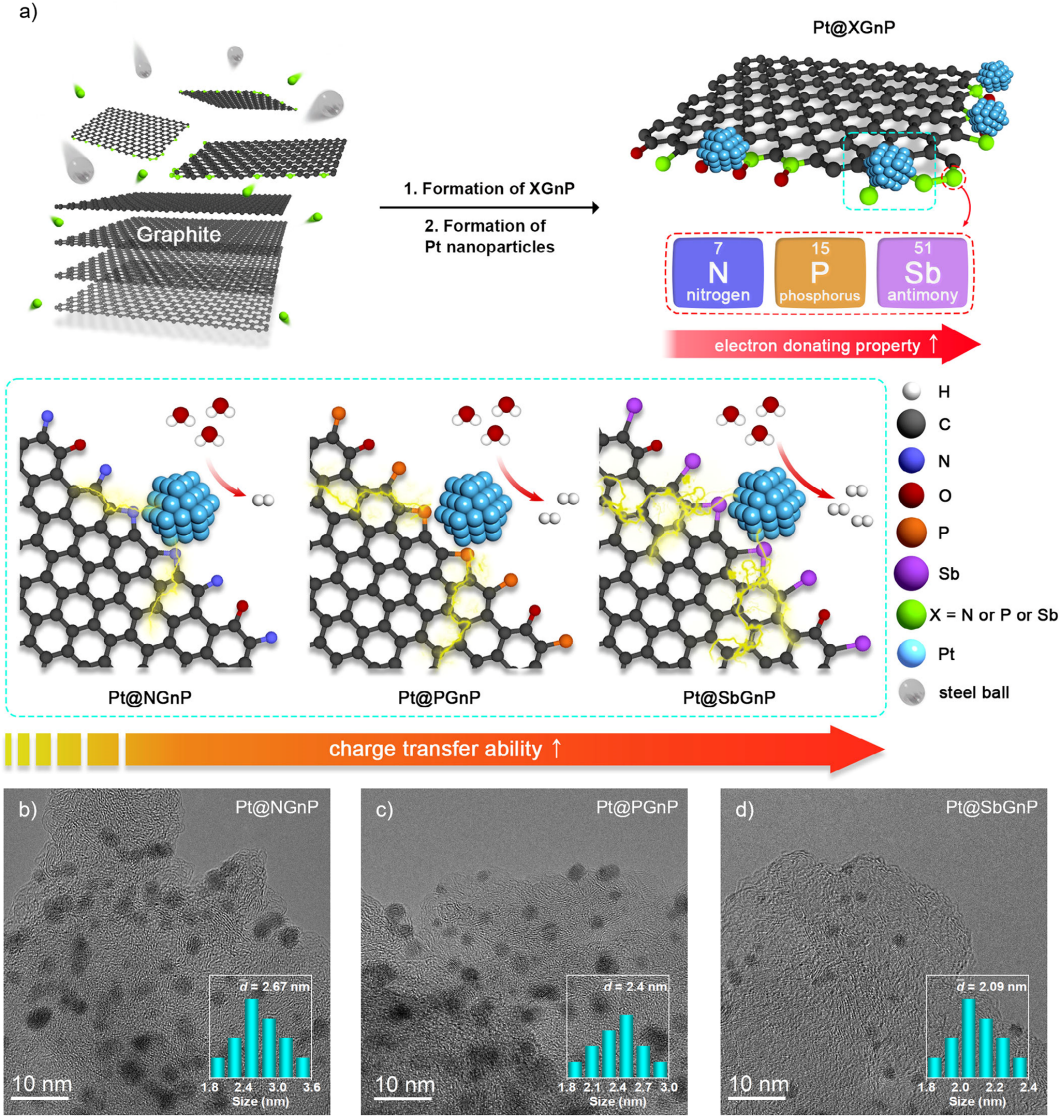
Preparation and morphology of Pt@XGnPs (X = N, P, or Sb). a) Schematic representation of the synthesis process. The yellow lines on Pt@XGnPs represent the degree of charge transfer between Pt NPs and XGnPs. b–d) HR‐TEM images of Pt@NGnP, Pt@PGnP, and Pt@SbGnP, respectively. The insets in (b–d) are the size distribution of Pt NPs.

A simple schematic illustration (**Figure** **1**a) represents the synthesis procedure of the Pt NPs impregnated group VA element‐doped graphitic nanoplatelets (Pt@XGnPs: X = N, P, or Sb) catalysts. The synthesis process commenced with the doping of heteroatoms (X = N, P, or Sb) onto GnPs (XGnPs) using a ball‐milling process with graphite, nitrogen gas (N_2_), red phosphorus (P), or antimony (Sb) ore. During this process, high‐speed traveling stainless steel balls deliver high impact energy to crack graphitic C−C bonds, generating active carbon species along the cracked edges.^[^ ^23^ ^]^ The activated edges of the GnPs react with N, P, or Sb, resulting in the edge‐selectively heteroatom‐doped GnPs. Doping heteroatoms on GnPs not only modified the electronic structure of pristine graphitic structure but also introduced anchoring sites for metal NPs. Consequently, the XGnPs could homogeneously anchor Pt precursors along the edges of the XGnPs. The Pt ions on the edges of XGnPs were then chemically reduced to zero‐valent metallic Pt NPs using sodium borohydride (NaBH_4_), forming Pt@XGnPs.^[^ ^24^ ^]^

To investigate the morphology of the Pt@XGnPs, high‐resolution transmission electron microscopy (HR‐TEM) and field emission scanning electron microscopy (FE‐SEM) images were obtained and analyzed. The TEM images clearly showed that small‐sized Pt NPs were uniformly deposited along the edges of the XGnPs. The average sizes of Pt NPs on Pt@NGnP, Pt@PGnP, and Pt@SbGnP were measured to be 2.67, 2.4, and 2.09 nm, respectively (Figure 1b–d). As previously discussed, doping with elements of larger atomic radius contributed to an increase in specific surface area, thereby facilitating the uniform and smaller‐sized distribution of Pt NPs. This trend was further supported by the Brunauer–Emmett–Teller (BET) analysis, which showed that SbGnP exhibited the highest specific surface area among the XGnPs (Figure S1, Supporting Information). In addition, HR‐TEM images confirmed the deposition of single Pt NPs along the XGnPs edges, while fast Fourier transform (FFT) patterns demonstrated a hexagonally ordered lattice of Pt (Figure S2, Supporting Information). FE‐SEM images revealed a round‐shaped morphology of Pt@XGnPs, in contrast to the flake‐type morphology previously observed (Figure S3, Supporting Information).^[^ ^24^ ^]^ Moreover, scanning transmission electron microscopy (STEM) images and corresponding energy‐dispersive X‐ray spectroscopy (EDS) elemental mapping images clearly revealed the presence of each doped element, C and Pt, further demonstrating the uniformity of the Pt NPs on XGnPs (Figure S4, Supporting Information). To quantify the Pt content, inductively coupled plasma optical emission spectroscopy (ICP‐OES) was conducted, revealing Pt loadings of 12.29, 11.74, and 8.47 wt.% for Pt@NGnP, Pt@PGnP, and Pt@SbGnP, respectively (Table S1, Supporting Information).

**Figure 2:**
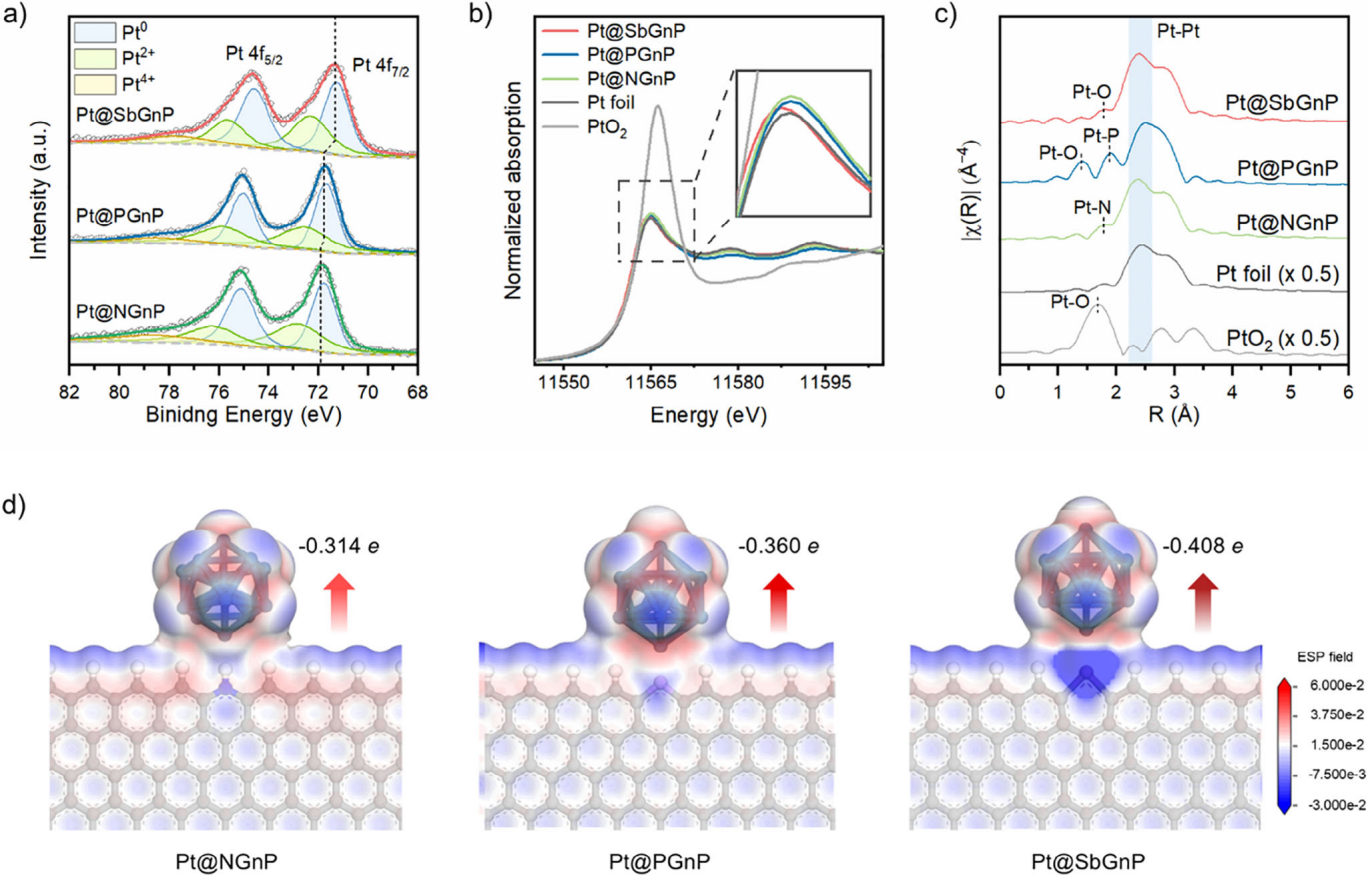
Characterization of structural and chemical states of the Pt@XGnPs. a) High‐resolution Pt 4f XPS spectra of Pt@NGnP, Pt@PGnP, and Pt@SbGnP. b) Pt L_3_‐edge XANES spectra of PtO_2_, Pt foil, Pt@NGnP, Pt@PGnP, and Pt@SbGnP. Inset: magnified white‐line profiles of Pt L_3_ edge XANES. c) EXAFS Fourier transforms of k^3^‐weighted Pt L_3_‐edge. d) Electrostatic potential maps around of the optimized Pt@NGnP, Pt@PGnP, and Pt@SbGnP. Blue and red surfaces represent positive and negative electrostatic potentials, respectively (isovalue = 0.001 a.u.).

X‐ray analytical techniques were employed to investigate the material properties and its metallic state. High‐power X‐ray diffraction (HP‐XRD) patterns were collected to analyze the crystal structures of the Pt@XGnPs (Figure S5, Supporting Information). The broad peak at a 2θ value of 25° for the Pt@XGnPs was assignable to the (002) graphitic lattice plane. The other sharp peaks of the Pt@XGnPs at 39.7°, 46.2°, and 67.6° corresponded to the (111), (200), and (220) of Pt crystal lattice planes, respectively. X‐ray photoelectron spectroscopy (XPS) was performed to investigate the elemental composition of the Pt@XGnPs. The XPS survey scan identified the presence of C, O, and Pt elements in the Pt@XGnPs (Figure S6, Supporting Information). Additionally, N, P, and Sb elements were included in the Pt@NGnP, Pt@PGnP, and Pt@SbGnP, respectively. The results confirmed doping heteroatoms and the deposition of Pt NPs on the Pt@XGnPs. XPS was then investigated to directly probe the surface charge state of Pt (**Figure** **2**a). The Pt@NGnP showed the highest binding energy of 71.9 eV in the Pt 4f spectra, while Pt@SbGnP exhibited a negatively shifted binding energy of 71.3 eV, revealing the enhanced charge transfer from SbGnP to Pt NPs.

The electronic structure of the Pt NPs was evaluated by X‐ray absorption near edge structure (XANES) measurements (Figure 2b). The white line (WL) of the Pt@XGnPs was located between the Pt foil and PtO_2_, indicating that the Pt NPs on Pt@XGnPs were partially oxidized. As the period of the doped heteroatoms increased, the WL intensity of Pt@XGnPs decreased, indicating a lower oxidation state of the Pt NPs.^[^ ^25^ ^]^ The highest WL intensity was found for Pt@NGnP, which was attributed to the relatively high electronegativity of the N‐doped GnP (NGnP), which facilitated the oxidation of Pt NPs. These results were in agreement with the XPS analysis (Figure 2a).

The k^3^‐weighted Fourier transform of the extended X‐ray adsorption fine structure (EXAFS) of the Pt L_3_‐edge of the Pt@XGnPs catalysts was obtained to demonstrate the coordination between the doped heteroatom of the Pt NPs and carbon supports (Figure 2c; Figures S7, S8, Supporting Information). All of the Pt@XGnPs exhibited distinct peaks corresponding to the Pt−O bond in PtO_2_ and the Pt–Pt bond in the Pt foil, respectively. P and Sb had more partial‐occupied p‐orbitals and vacant d‐orbitals than N, allowing them to accommodate additional chemical bonds to form more stable structures.^[^ ^16^ ^]^ During the synthesis process, P or Sb doped on the GnPs (PGnP or SbGnP) were partially oxidized, resulting in the formation of stable Pt–O coordination at 1.4 and 1.7 Å, respectively.^[^ ^16^, ^26^ ^]^ In addition, the peaks observed at 1.7 and 1.9 Å could be attributed to Pt–N and Pt–P bonding in Pt@NGnP and Pt@PGnP, respectively.^[^ ^27^ ^]^ Wavelet transform (WT) analysis of the EXAFS spectra revealed Pt–Pt coordination in the Pt@XGnPs, with intensity maxima near R = 2.7 Å and k ≈ 11 Å^−1^, similar to Pt foil (Figure S9, Supporting Information). Therefore, these findings agreed with the XPS analysis (Figure 2a), and the overall structural analysis suggests that the extent of electron transfer from the XGnPs to the Pt NPs varied depending on the type of dopant element.

To elucidate the enhanced charge transfer performance of SbGnP compared to NGnP and PGnP, we performed density functional theory (DFT) calculations. A total of seventeen XGnP models of five NGnPs, six PGnPs, and six SbGnPs structures were constructed depending on the functional groups (Figure S10, Supporting Information, see computational details in Supporting Information). Upon the adsorption of Pt NP onto each element‐doped region (Figure S11, Supporting Information), the total charge of Pt NP was calculated (Figure S12, Supporting Information). The Pt NPs exhibited negative charges for all models, indicating electron transfer occurred from XGnPs (X = N, P, or Sb) to Pt NP. Among our models, the NH–, P–, and Sb–functionalized structures exhibited the largest charge transfer within each similar type of dopant group. Notably, the magnitude of charge transfer followed the order of Pt@SbGnP (Sb), Pt@PGnP (P), and Pt@NGnP (NH) (i.e., −0.408 e, −0.360 e, and −0.314 e), consistent with the experimental XPS (Figure 2a) and XANES (Figure 2b) results.

Additionally, the electrostatic potential (ESP) maps of all models are presented in Figure S13 (Supporting Information), and the Pt@NGnP (NH), Pt@PGnP (P), and Pt@SbGnP (Sb) models are selectively shown in Figure 2d. It is clearly seen that Pt@SbGnP strongly induced positively charged on Sb atom, which is associated with the lower electronegativity of Sb relative to N and P. The DFT modeling was constructed based on experimentally obtained XPS data. The GnP structure was classified into two types depending on the edge configuration: zigzag and armchair. Typically, when the main peak of the C 1s XPS spectrum appeared around 284 eV, it corresponded to a zigzag edge, whereas a peak around 283 eV indicated an armchair edge.

In the synthesized Pt@XGnP series, the main C 1s peak was consistently observed at 284 eV, indicating zigzag edge structures were predominant (Figure S14, Supporting Information).^[^ ^28^, ^29^ ^]^ In the case of Pt@PGnP, the P 2p spectrum exhibited peaks corresponding to both P–O and P–C bonds. Among them, the P–C peak demonstrated a larger area ratio, suggesting a higher likelihood of Pt–P bond formation.^[^ ^30^ ^]^ For Pt@SbGnP, the Sb 3d spectrum was analyzed to compare the relative peak areas of the Sb^3+^ and Sb^5+^ oxidation states, and Sb^3+^ was found to be the dominant species. Notably, Sb^3+^ tended to form bonds into metallic Sb in the zigzag configuration, supporting the formation of Pt–Sb bonds as the predominant structure (Figure S15, Supporting Information).^[^ ^31^ ^]^

Raman spectroscopy provided important information about the presence of defects and electron interactions in the materials, and these properties had a significant impact on the peak shift related to electron transfer. In particular, the migration of the G band associated with sp^2^ hybridization reflected structural changes caused by functionalization or defects.^[^ ^32^ ^]^ The electron density was modulated depending on the heteroatoms (N, P, or Sb) introduced at the edges of the graphitic framework. Among them, Sb, which had the highest electropositivity, induced charge transfer to the Pt NPs most effectively. In addition, the lower the G band position, the better the electron transfer from the support to the metal (Figure S16, Supporting Information).^[^ ^33^ ^]^

**Figure 3:**
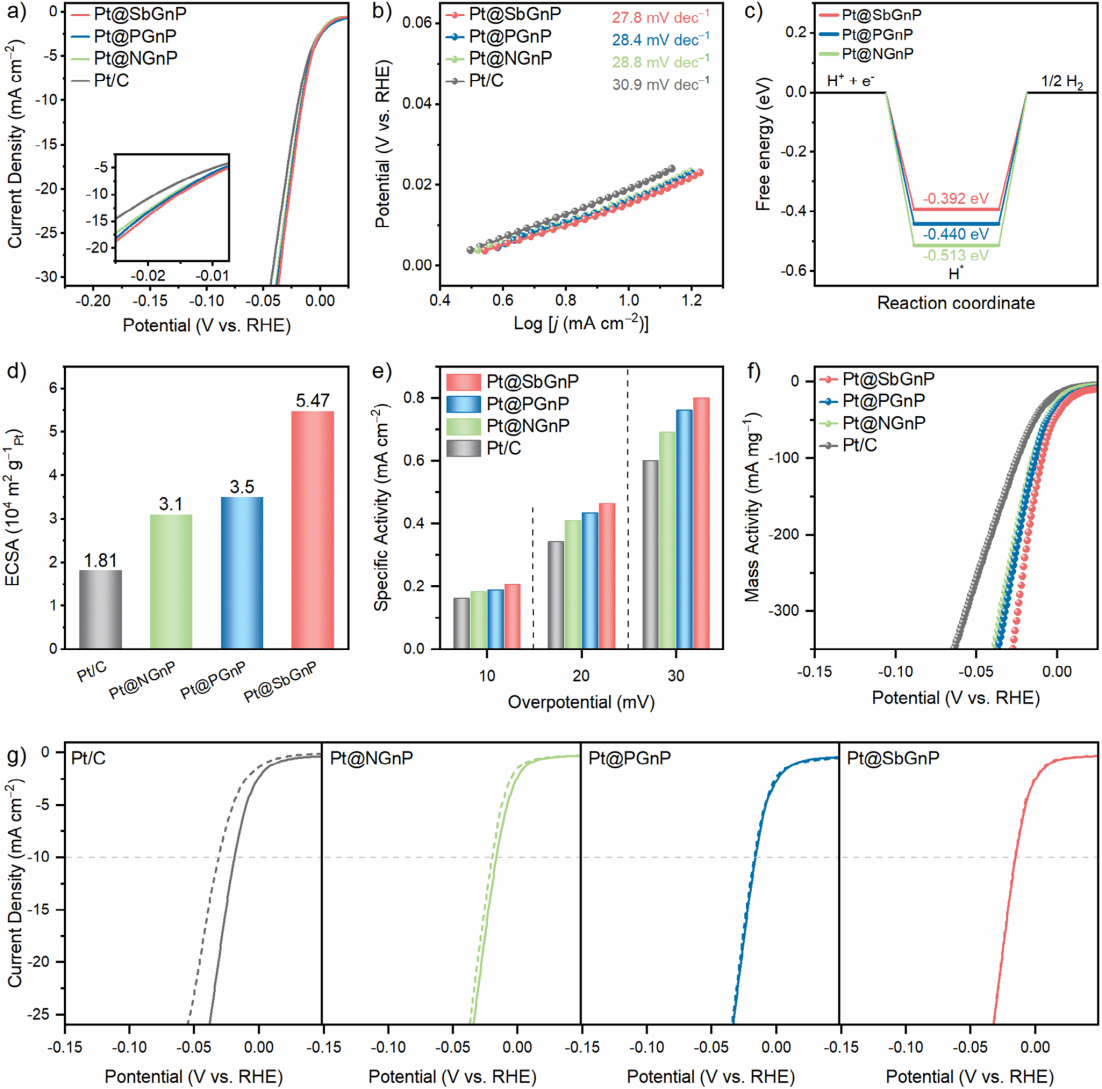
Half‐cell electrocatalytic performance of Pt@XGnPs. a) HER polarization curves of Pt/C and Pt@XGnPs. Inset: magnified polarization curves. b) Tafel plots obtained from the polarization curves. c) H‐adsorption free energy diagram of Pt@NGnP, Pt@PGnP, and Pt@SbGnP, respectively. d) Comparison of the ECSA calculated from capacitive current. e) Specific activities at different overpotentials (10, 20, and 30 mV) in N_2_‐saturated in 0.5 m aq. H_2_SO_4_. f) Mass activities of electrocatalysts in N_2_‐saturated in 0.5 m aq. H_2_SO_4_. g) Polarization curves for Pt/C and Pt@XGnPs catalysts before and after 10 000 cycles.

The electrocatalytic performance toward the hydrogen evolution reaction (HER) of Pt@NGnP, Pt@PGnP, and Pt@SbGnP was evaluated using a standard three‐electrode system in N_2_‐saturated 0.5 m aq. H_2_SO_4_. For comparison, commercial Pt/C, and the as‐prepared XGnPs (X = N, P, or Sb) were also investigated under the same conditions. The Pt@XGnPs showed superior electrocatalytic HER activity to the XGnPs supports (Figure S17, Supporting Information). Pt@SbGnP showed the lowest overpotential (15.3 mV), outperforming Pt@NGnP (16.5 mV), Pt@PGnP (16.0 mV), and commercial Pt/C (19.0 mV) (**Figure** **3**a). Compared to recently reported Pt NPs‐based catalysts, the Pt@XGnPs exhibited comparable or even superior performance (Figure S18 and Table S3, Supporting Information). The Tafel slopes for Pt@NGnP, Pt@PGnP, and Pt@SbGnP were 28.8, 28.4, and 27.8 mV dec^−1^, respectively, and were all lower than that of Pt/C (30.9 mV dec^−1^), indicating faster kinetics (Figure 3b).^[^ ^34^ ^]^

Electrochemical impedance spectroscopy (EIS) analysis revealed that the charge transfer resistance (R_ct_) at an overpotential of 10 mV for Pt@XGnPs was lower than that of Pt/C (Figure S19, Supporting Information). Among the Pt@XGnPs catalysts, Pt@SbGnP exhibited the smallest R_ct_ of 1.67 Ω cm^2^, demonstrating better interfacial charge transfer kinetics.^[^ ^35^ ^]^ The enhanced HER activity was attributed to the high conductivity of the XGnP supports and efficient electron transfer aligned with the periodic trend of the doped elements. The exchange current density (*J_0_*) values for Pt@NGnP, Pt@PGnP, Pt@SbGnP, and Pt/C were 2.61, 2.65, 2.70, and 2.40 mA cm^−2^, respectively (Figure S20, Supporting Information). This trend corroborated the observed overpotential and R_ct_ values. Overall, HER activity followed the order: Pt/C < Pt@NGnP < Pt@PGnP < Pt@SbGnP. The outstanding performance of Pt@SbGnP was attributed to Sb's low electronegativity and favorable electronic properties, which enhanced electron transfer to the Pt NPs and strengthened metal–support interactions.

To explore the enhanced electrocatalytic activity of Pt@SbGnP compared to Pt@NGnP and Pt@PGnP, we calculated the hydrogen adsorption Gibbs free energy (Δ*G_H_*) of the Pt@XGnP models (Figure S21, Supporting Information). The (Δ*G_H_*) values followed the order of Pt@NGnP, Pt@PGnP, and Pt@SbGnP (i.e., −0.513, −0.440, and −0.392 eV), which was consistent with the trend in experimental overpotential (Figure 3c). Pt@SbGnP exhibited weaker hydrogen bonding compared to Pt@NGnP and Pt@PGnP, indicating more favorable dissociation of the Pt–H bond, to release H_2_ gas. To this end, it was theoretically suggested that the Pt@SbGnP exhibited enhanced electrocatalytic activity due to the weak hydrogen adsorption on electron‐rich Pt NPs, resulting from the smaller electronegativity of the doped Sb.^[^ ^36^ ^]^

The electrochemical surface area (ECSA) of the samples was further studied by underpotential deposition (UPD) of copper (Figures S22, S23, Supporting Information). The ECSA of Pt@SbGnP was ≈49.6 m^2^ g^−1^_Pt_, surpassing that of Pt@NGnP (36.5 m^2^ g^−1^_Pt_), Pt@PGnP (36.0 m^2^ g^−1^_Pt_), and Pt/C (21.9 m^2^ g^−1^_Pt_). This enhancement was attributed to the XGnPs support, which enabled the formation of well‐dispersed, small‐sized Pt NPs, offering more active sites than Pt/C. Additionally, double‐layer capacitance (C_dl_) was measured using cyclic voltammetry (CV) in the range of 0.1–0.3 V (vs RHE). Pt@SbGnP exhibited a C_dl_ value of 167 mF cm^−2^, exceeding those of Pt@NGnP (137 mF cm^−2^), Pt@PGnP (148 mF cm^−2^), and Pt/C (131 mF cm^−2^) (Figure 3d; Figure S24, Supporting Information). The superior ECSA of Pt@SbGnP was attributed to the large atomic radius of Sb, which provided a broader surface area for the carbon support compared to N– or P–doped supports. As a result, the Cu‐UPD and C_dl_ measurements confirmed that Pt@SbGnP exhibited nearly twice the ECSA compared to Pt/C and other Pt@XGnPs, reinforcing its exceptional catalytic performance.

The turnover frequency (TOF) values of Pt@XGnPs and Pt/C were derived from the number of active sites, as determined by Cu‐UPD measurements.^[^ ^37^ ^]^ The Pt@SbGnP showed a large TOF of 0.85 H_2_ s^−1^ at an overpotential of 25 mV, whose value was better than Pt@NGnP (0.70 H_2_ s^−1^), Pt@PGnP (0.79 H_2_ s^−1^), Pt/C (0.60 H_2_ s^−1^), and other recently reported electrocatalysts (Figure S25, Supporting Information). The polarization curves normalized by ECSA offered insights into the intrinsic catalytic activity of each catalyst. All of the Pt@XGnPs exhibited higher specific activity than Pt/C across the entire overpotential range (Figure 3e). Among the catalysts, Pt@SbGnP demonstrated the highest specific activity, establishing itself as the most efficient and active HER catalyst, capable of adsorbing protons at the fastest rate, or producing hydrogen with the greatest efficiency.

The mass activity, which is directly related to the cost of catalyst in practice, was explored by normalizing the LSV curves with the Pt loading of each catalyst (Figure 3f). Pt@SbGnP demonstrated the highest mass activity, highlighting the significant advantages of doping the high‐period element Sb onto the carbon support, to enhance both catalytic performance and cost efficiency. To identify the active sites of the catalysts, a poisoning test was conducted by adding thiocyanate ions (^–^SCN), which selectively poison metal catalytic sites. Upon the addition of ^–^SCN, the catalytic activity of the Pt@XGnPs sharply decreased, confirming that the Pt NPs on Pt@XGnPs served as the active sites for the HER (Figure S26, Supporting Information).^[^ ^38^ ^]^

The long‐term stability of Pt@XGnPs was evaluated through accelerated degradation tests (ADT) (Figure 3g). The polarization curves with dashed lines were obtained after 10 000 continuous CVs. While Pt/C retained only 60.9% of its initial activity, the Pt@XGnPs exhibited excellent durability. Notably, Pt@SbGnP maintained 97.5% of its initial performance, demonstrating remarkable long‐term stability (Figure S27, Supporting Information). To further assess durability, chronoamperometry (CA) scans at an applied potential of 20 mV for 40 hours were acquired. All of the Pt@XGnPs showed minimal current loss, outperforming Pt/C in stability (Figure S28, Supporting Information). After the stability test, the morphology of the Pt@XGnPs remained intact, devoid of any discernible Pt aggregates (Figure S29, Supporting Information). The superior durability of the Pt@XGnPs, especially Pt@SbGnP, is attributed to their strong metal–support interactions. SbGnP's larger surface area enabled the uniform dispersion of smaller Pt NPs, while its unique electronic structure provided stronger binding with Pt than N– or P–doped supports. This synergy enhanced both the stability and catalytic performance of Pt@SbGnP over Pt/C and other Pt@XGnPs.

**Figure 4:**
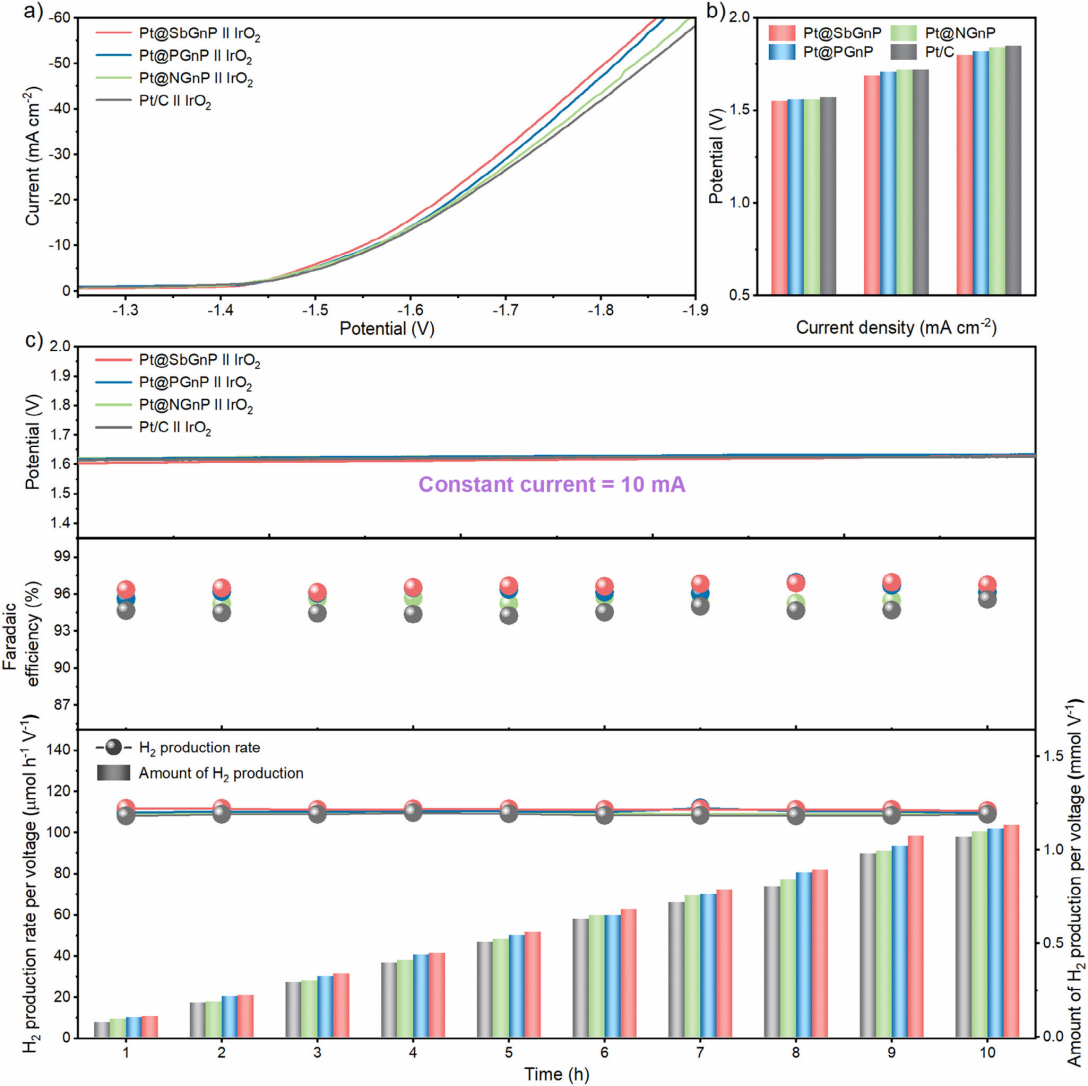
Overall water‐splitting system of Pt@XGnPs. a) Polarization curves of Pt/C, Pt@NGnP, Pt@PGnP, and Pt@SbGnP. b) Comparison of current density at 1.7, 1.8, and 1.9 V. c) Measurement of cell potential change, Faradaic efficiency, hydrogen production rate, and amount of hydrogen production per voltage at specific current of 10 mA.

Inspired by the high efficiency and durability of Pt@XGnPs in the half‐cell tests for HER, two‐electrode full cell systems were assembled, and the overall water splitting system was evaluated in N_2_‐saturated 0.5 m aq. H_2_SO_4_. Pt@XGnP and Pt/C catalysts were coated on carbon paper (CP) by electrospray to fabricate a cathode for water splitting, and commercial iridium oxide (IrO_2_) catalyst coated on CP was employed as the anode. The polarization curves of Pt/C and Pt@XGnPs showed the same trends as the half‐cell system and confirmed that Pt@SbGnP had the best electrochemical performance (**Figure** **4**a). At current densities corresponding to cell voltages of 1.7, 1.8, and 1.9 V, the performance followed the order: Pt/C < Pt@NGnP < Pt@PGnP < Pt@SbGnP (Figure 4b). When a cell voltage of 1.9 V was applied, the current density of Pt@SbGnP was 68.2 mA cm^−2^, which was significantly higher than that of Pt/C (58.4 mA cm^−2^), Pt@NGnP (60.9 mA cm^−2^), and Pt@PGnP (66.0 mA cm^−2^).

A gas chromatography (GC) instrument was connected to a closed water‐splitting system to evaluate the actual amount of gas products and the corresponding Faradaic efficiency. All analytical results were obtained by applying a constant current of 10 mA and measuring the amount of hydrogen produced each hour (Figure 4c). A current of 10 mA was achieved at only 1.59 V for Pt@SbGnP and 1.61 V for commercial Pt/C. After 10 hours of durability testing, the systems with Pt@XGnPs exhibited no significant degradation, with cell voltages remaining similar to the initial state. Faradaic efficiency of catalysts was measured at a constant current of 10 mA, indicative of the percentage of the total charge used in the reaction that was used to produce hydrogen. The Pt@SbGnP catalyst showed a Faradaic efficiency of 96.6%, which was higher than Pt@NGnP (95.5%), Pt@PGnP (96.3%), and Pt/C (94.7%). The amount of H_2_ production of Pt@XGnPs was significantly higher than that of Pt/C and increased in the order Pt@NGnP < Pt@PGnP < Pt@SbGnP. This was attributed to the improved electron transfer to the Pt NPs as the higher period of the doped X (X = N, P, or Sb) element increased, which positively influenced the overall electrochemical performance.

**Figure 5:**
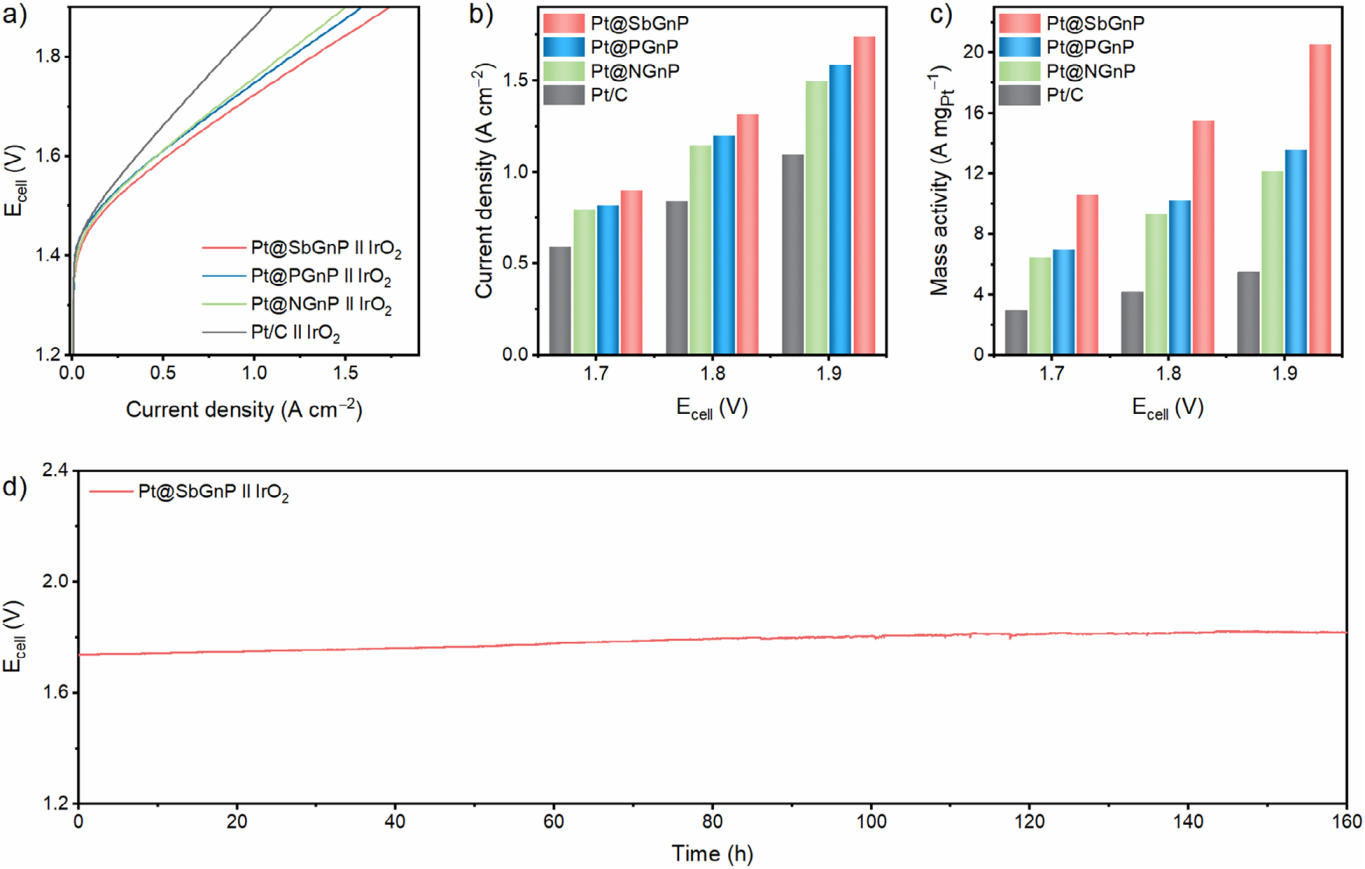
PEMWE performance of Pt@XGnPs. a) Polarization curves of Pt/C and Pt@XGnPs. b) Current densities and c) mass activities at different operating voltages. d) Stability test of PEMWE at the specific current density of 1 A cm^−2^.

The catalytic potential of the developed Pt@XGnPs was evaluated under PEMWE tests, simulating industrial conditions in the laboratory. A membrane electrode assembly (MEA) was fabricated using the electrospray technique, ensuring uniform coating on both the membrane and substrate (Figure S30, Supporting Information). For the cathode, commercial Pt/C and the synthesized Pt@XGnPs were utilized, while commercial IrO_2_ served as the anode catalyst. To attain a current density of 1 A cm^−2^, the Pt@XGnP catalysts required markedly lower cell voltages compared to commercial Pt/C (1.857 V), with Pt@NGnP (1.759 V), Pt@PGnP (1.748 V), and Pt@SbGnP (1.724 V) demonstrating superior performance (**Figure** **5**a). Furthermore, Pt@SbGnP demonstrated the best performance in terms of current density at equivalent voltages, achieving 1.740 A cm^−2^ at 1.9 V, which surpassed the performance of Pt/C (1.098 A cm^−2^), Pt@NGnP (1.496 A cm^−2^), and Pt@PGnP (1.586 A cm^−2^) (Figure 5b). These results were consistent with the trends observed in prior half‐cell and two‐electrode system tests, thereby confirming that the Pt@XGnPs demonstrate significantly enhanced performance compared to commercial Pt/C. In addition, at 1.9 V, all of the Pt@XGnPs demonstrated significantly higher mass activity compared to Pt/C (Figure 5c). Notably, Pt@SbGnP achieved nearly threefold the performance of Pt/C. The recorded mass activity values were as follows: Pt/C (5.49 A mg_Pt_^−1^), Pt@NGnP (12.17 A mg_Pt_^−1^), Pt@PGnP (13.56 A mg_Pt_^−1^), and Pt@SbGnP (20.53 A mg_Pt_^−1^). The chronopotentiometry curve, measured at 80 °C under a current density of 1 A cm^−2^, confirmed that the Pt@SbGnP catalyst exhibited outstanding stability and retained its performance for nearly a week (Figure 5d). After the stability test, supplementary XPS and XRD analyses were performed on the Pt@SbGnP catalyst. The results confirmed that both the chemical composition and crystalline structure remained unchanged, thereby further verifying the compositional and structural stability of the catalyst under prolonged operation (Figures S31, S32, Supporting Information).

## 3 Conclusion

In summary, we have demonstrated that high‐period element doping of carbon supports can play a pivotal role in enhancing electronic metal–support interaction (EMSI) and catalytic performance in acidic hydrogen evolution reactions (HER). A comparative study of group VA element‐doped graphitic nanoplatelets (XGnPs; X = N, P, or Sb) was conducted. The result revealed that as the period of the dopant heteroatom increased, it significantly promoted charge transfer to Pt NPs via strong Pt–X bonding, as evidenced by X‐ray spectroscopies, DFT calculations, and Raman analyses. Among the doped heteroatoms, Sb possesses high electropositivity, larger atomic radius, and unique orbital characteristics. These properties facilitated optimal Pt dispersion, boosted the electrochemical surface area, and enhanced the ultimate strength of EMSI. These effects resulted in remarkable HER performance with an overpotential of only 15.3 mV, excellent stability over 10 000 cycles, and a high Faradaic efficiency of 96.6%. Furthermore, the Pt@SbGnP catalyst achieved industrially relevant performance in a practical PEMWE, delivering 1 A cm^−2^ at a voltage as low as 1.724 V with sustained operation over one week. This study highlights a heteroatom‐periodicity‐driven strategy for tuning metal–support interactions and provides new design principles for developing efficient, durable, and cost‐effective HER electrocatalysts for next‐generation hydrogen production.

## Author Contributions

J.B. supervised the project. I.J. and D.K. contributed to material synthesis and catalyst improvement ideas. J.B. and S.L. designed and conducted the electrolyzer tests, performed catalyst characterization, analyzed the data, and wrote the original manuscript. N.K. carried out catalyst synthesis, characterization, and half‐cell tests. S.L. and S.K. conducted DFT calculations and provided theoretical insights. J.S., H.N., J.J., and C.L. contributed to data discussions.

## Conflict of Interest

The authors declare no conflict of interest.

## Supporting information



Supporting Information

## Data Availability

The data that support the findings of this study are available from the corresponding author upon reasonable request.
