# Supplementary material for: High‐Period Element Doping as a Key Driver of Hydrogen Evolution in a Proton Exchange Membrane Water Electrolyzer
Source: Small. 2025 Aug 8;21(39):e07086. doi: 10.1002/smll.202507086 (PMC12490176; doi:10.1002/smll.202507086)
Supplement: Supplementary file 1 — Supporting Information [file SMLL-21-e07086-s001.docx]

Supporting Information

**High-period element doping as a key driver of hydrogen evolution in a proton exchange membrane water electrolyzer**

**Methods**

**Synthesis of XGnP (X=N, P, and Sb)**

NGnP, PGnP and SbGnP were prepared by ball-milling in the presence of pristine graphite (5.0 g, Alfa Aesar, natural graphite, 100 mesh (< 150 μm), 99.9995% metals basis, lot# 14735) with a respective reactant. To prepare NGnP, PGnP, and SbGnP, the graphite together with nitrogen gas (10 bar), red phosphorus (20.0 g), and Sb (5.0 g) were, respectively, charged in a planetary ball-mill container (250 mL) together with stainless steel balls (500.0 g, diameter: 5 mm). After agitating at a rate of 500 rpm for 48h, unreacted reactants and remnant metallic impurities were removed by repeatedly washing with concentrated HCl, and the resultant products were thoroughly washed with distilled water. The final dark black XGnP powders were collected by filtration, subjected to further Soxhlet extraction with concentrated HCl and water. Finally, XGnPs were freeze-dried at −120 ℃ under reduced pressure (0.1 mmHg) for 48 h.

**Synthesis of Pt NPs on XGnPs (Pt@XGnPs)**

A round bottom flask containing 200 mg of each XGnP in 100 mL of deionized water (DI water) was sonicated for 1 h to disperse the powder. 50 mg of chloroplatinic acid hydrate (H_2_PtCl_6_·H_2_O) dispersed in 50 mL of DI water was added into the reactor, while vigorously stirring for 24 h. For forming Pt NPs, 100 mL of 0.1 M sodium borohydride (NaBH_4_) solution was slowly added to the reactor while vigorously stirring. After filtration and washing with DI water, Pt NPs deposited on XGnP (Pt@XGnP) catalysts were obtained. Then, Pt@XGnPs were freeze-dried at −120 ℃ under reduced pressure (0.1 mmHg) for 48 h.

**Electrochemical Measurement**

The electrochemical performance of each catalyst was measured in a typical three-electrode system. The used working electrode, reference electrode and counter electrode were a rotating ring-disk electrode (RRDE with a diameter of 4 mm), an Ag/AgCl (saturated KCl) and a graphite rod, respectively. The ink of each catalyst was prepared by dispersing in isopropyl alcohol (IPA, 980 μL) containing Nafion (20 μL, 5 wt% in a mixture of lower aliphatic alcohol and water, Aldrich Chemical Inc.). Followed by sonication in an ice bath for 1 h, the catalyst ink of 18 μL with a catalyst mass of 90 μg was drop cast onto RRDE and dried at room temperature for 10 min, forming a flat film. Linear sweep voltammetry (LSV) was performed in 0.5 M aq. H_2_SO_4_ at a 5 mV s^−1^ scan rate and 1600 rpm at room temperature. The solution resistances (R_s_) in the electrolyte were measured to 3 Ω by Nyquist plots. A 95% Ohmic drop (iR) correction is applied to all measured polarization curves. After the calibration of the reference electrode, all potential was adjusted relative to RHE (Figure S29). Electrochemical impedance spectroscopy (EIS) was conducted at a frequency range of 0.1 to 105 Hz. Cyclic voltammetry (CV) was carried out for a durability test with a scan rate of 100 mV s^−1^ for 10,000 cycles. Chronoamperometry (CA) was done at an overpotential of 20 mV with iR-compensation applied. The underpotential deposition (UPD) of copper (Cu) measurements were conducted in a 0.5 M aq. H_2_SO_4_ solution. Prior to Cu stripping in the absence and presence of 5 mM CuSO_4_ at a scan rate of 10 mV s^−1^, a UPD layer was formed by polarizing the electrode at 0.314 V for 100 s. The poisoning experiment was performed in 0.5 M aq. H_2_SO_4_ by adding 10 mM NaSCN, while conducting the chronoamperometry (CA) measurement.

**Material Characterization**

Scanning electron microscopy (SEM) data were collected using Field Emission−Scanning Electron Microscope (FE−SEM; SU7000) (Hitachi, Japan) equipment with an accelerating voltage of 10 kV. X−ray diffraction (XRD) analysis was performed by High−Power X−Ray Diffractometer (HPXRD; D/MAX 2500 V/PC) (Rigaku, Japan) with a Cu−Kα anode (40 kV, 200 mA, λ=1.54056 Å). N_2_ adsorption-desorption isotherms were measured using a BELSORP-max at 77 K. The surface area was determined by the Brunauer-Emmett-Teller (BET) method. High−resolution transmission electron microscopy (HR−TEM) images were taken with a JEM−2100F microscope (JEOL, Japan) with an accelerating voltage of 200 keV. To prepare the TEM specimens, the catalysts were dispersed in acetone solution and dropped on holey carbon TEM grid (200 Mesh Copper Grid) (Ted Pella, USA). Subsequently, the grid was dried in a vacuum oven at 70 °C. X−ray photoelectron spectroscopy (XPS) was measured with an X−ray Photoelectron Spectrometer (Thermo Fisher K−alpha, UK). Electrocatalytic performance was measured by electrochemical workstation (CompactStat.h) (Ivium, Netherlands). X-ray absorption fine spectra of the prepared catalysts were collected in the transmission mode using ionization detectors (Oxford) at the Pohang Accelerator Laboratory (PAL). The X-ray absorption spectra for the Pt L edge was acquired at room temperature using beamline 6D of PAL, where the X-ray energies from the EXAFS analysis were calibrated with Pt foil. Background subtraction, normalization and Fourier transformation (FT) were performed using standard procedures with the ATHENA program in the IFEFFIT software package. k^3^-weighted EXAFS oscillations ranging from 3.0 to 10.5 Å were Fourier transformed to obtain a radial distribution function.

**Full water splitting electrode measurements**

For the two-electrode system, ink consisting of each sample (30 mg), water, isopropanol, and ionomer was prepared, and the ionomer-to-carbon (I/C) ratio of the ink was 0.6. The ink was then subjected to sonication in an ice bath for 30 min and rotation using a swing planetary mixer (HSPM-1.5) to obtain a homogeneous dispersion. Anode and cathode electrodes were prepared using the electrospray method. At first, the sample dispersion solution was loaded into a plastic syringe equipped with a 30-gauge stainless steel hypodermic needle. The syringe was connected to a high-voltage power supply (ESN-HV30), and the solution was delivered at a constant flow rate of 75 μL min^−1^ using a syringe pump (KD Scientific Model 220). A voltage of approximately 6.0 kV was applied at a height of 8.5 cm and deposited on a 1×1 cm^2^ area of carbon paper (CP) according to the Pt content of each sample. Consequently, each Pt@XGnP was loaded onto the CP in the same amount as 20 wt% Pt/C and were used as a cathode, while IrO_2_ was used as an anode.

**PEMWE measurements**

The membrane electrode assembly (MEA) was prepared using a combination of the catalyst-coated membrane (CCM) and catalyst-coated substrate (CCS) methods. For the anode, commercial IrO_2_ was used as the catalyst, while for the cathode, commercial Pt/C (20 wt%) and Pt@XGnPs (X = N, P, or Sb) were employed. All catalysts were first dispersed in a mixture of isopropanol, DI water, and Nafion solution, and the mixture was ultrasonicated for 1 hour to produce homogeneous ink. A titanium fiber felt was used as the porous transport layer (PTL) for the anode, and carbon paper served as the gas diffusion layer (GDL) for the cathode. The prepared anode and cathode inks were then directly coated onto the Nafion 115 membrane, PTL, and GDL using an electrospray technique. The catalyst mass loading was controlled at 1.0 mg cm^−2^ for both the anode (IrO_2_) and cathode (Pt/C and Pt@XGnPs). The MEA, along with the GDL and PTL, was hot-pressed at 120 ℃ and 2 MPa for 3 min. The PEMWE was tested at 80 ℃ using distilled water as the electrolyte. A peristaltic pump was employed to circulate the electrolyte at a flow rate of 20 mL min^−1^. All voltage measurements recorded during the PEMWE tests were obtained without iR correction.

**Computational details**

*Molecular modeling*: Zigzag graphitic nanoribbon models were used as GnPs, and each dopant (N, P, or Sb) was introduced in the form of functional groups consisting of O and H (Figure S10).^[1-6]^ A Pt_13_ icosahedron nanocluster model was used as the Pt NP and was optimally placed on the top of each dopant functional group for the Pt@XGnP models (Figure S11). For the adsorption site of hydrogen atom on Pt NP, the top Pt atom was selected considering steric hindrance and hydrogen accessibility (Figure S21).

*Density functional theory (DFT) calculations*: DFT calculations were performed using the CASTEP program.^[7]^ We employed the ultrasoft pseudopotential and the generalized gradient approximation (GGA) with the Perdew-Burke-Ernzerhof (PBE) functional.^[8]^ The Tkatchenko–Scheffler (TS) method was utilized for dispersion correction.^[9]^ Electronic wave functions were expanded in a plane-wave basis set with a kinetic energy cutoff of 340 eV. The convergence criteria for energy, force, and displacement were set to 2 × 10^−5^ eV/atom, 0.05 eV/Å, and 0.002 Å, respectively. The Brillouin zone was integrated using a gamma-centered k-point grid with a separation of 0.04 Å^−1^. To analyze atomic charges, we employed the Hirshfeld method.^[10]^ The Gibbs free energy of adsorbed atomic hydrogen was calculated as follows,

|  | ${\Delta G}_{H}={\Delta E}_{H}+\Delta ZPE-T\Delta S$ |  |
| --- | --- | --- |

where ${\Delta E}_{H}$ is the adsorption energy of the hydrogen atom on the Pt@XGnPs, $\Delta ZPE$ is the difference of the zero-point energy between the adsorbed hydrogen and hydrogen in the gas phase, and $\Delta S$ is the entropy change of hydrogen adsorption whose adsorbed state is negligible.





**Figure S1.**  (a) Brunauer-Emmett-Teller (BET) adsorption-desorption isotherm of XGnPs (X = N, P, or Sb) measured at 77 K. (b) Calculated specific surface areas.


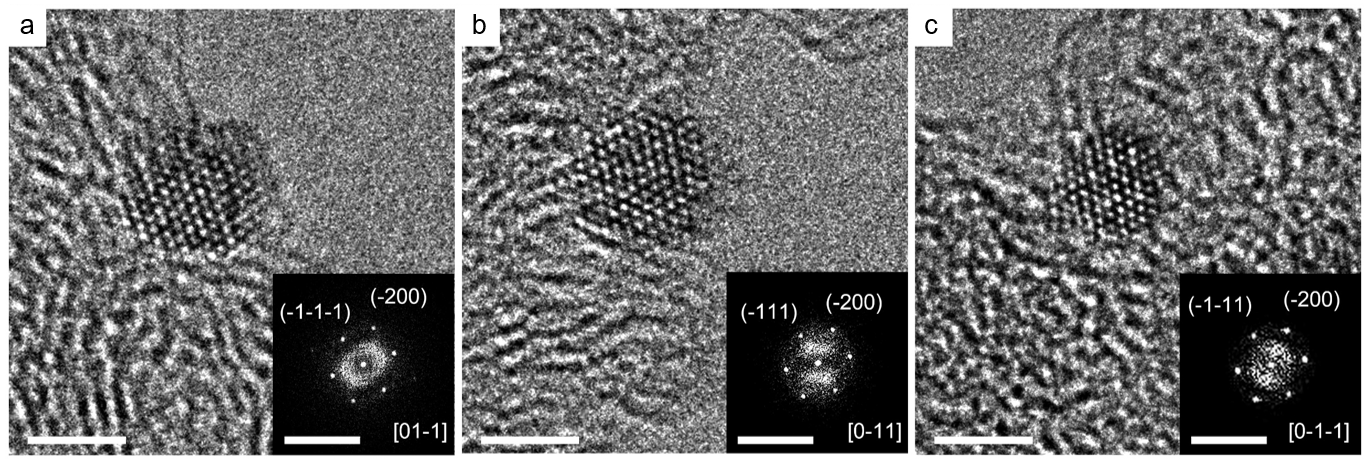


**Figure S2.** HR-TEM images focused on an Pt nanoparticles (NPs) on: (a) Pt@NGnP; (b) Pt@PGnP; (c) Pt@SbGnP, respectively, showing the high crystallinity of the Pt NPs with compact packing in the lattice. Scale bars in (a-c): 10 nm.


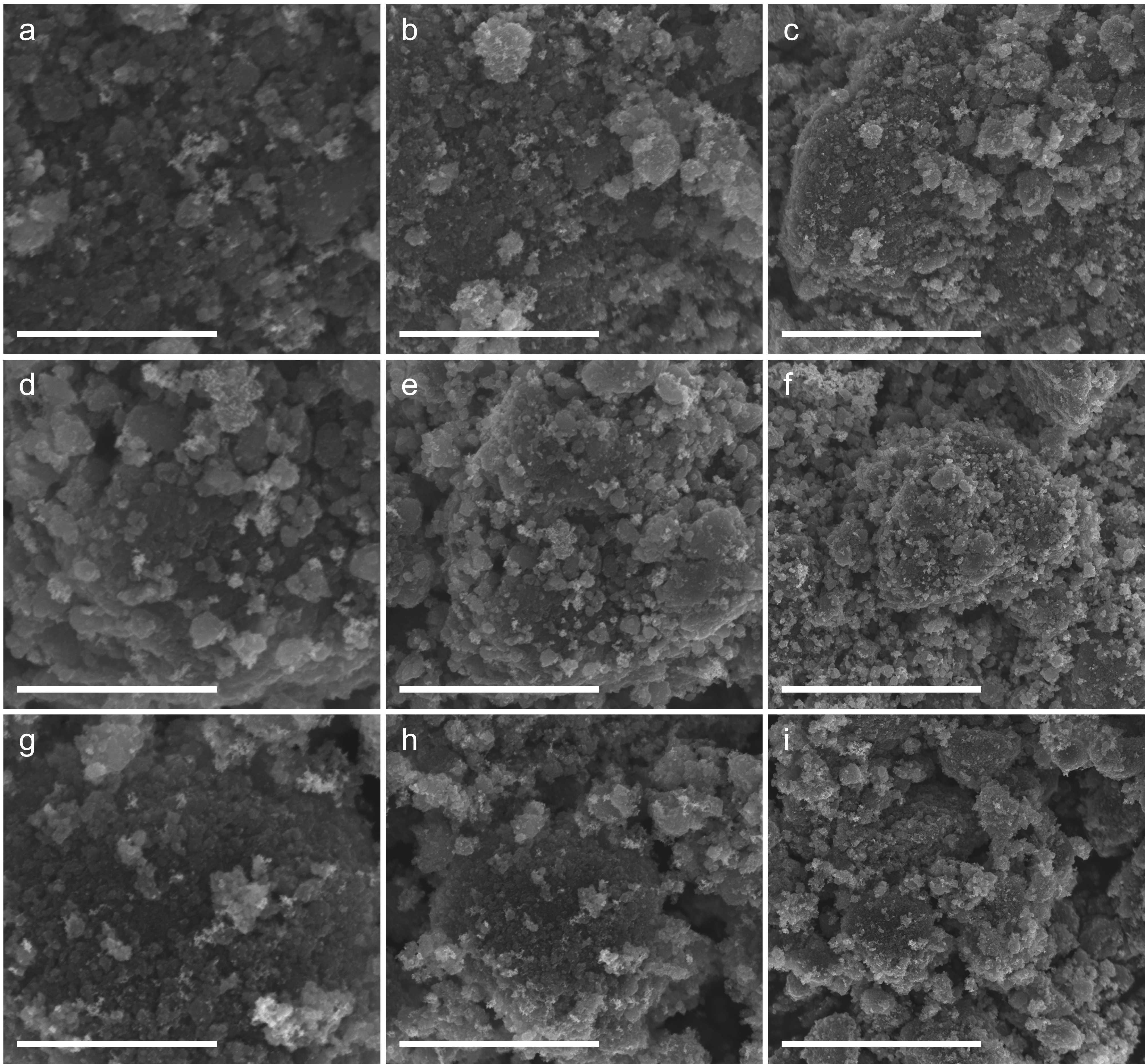


**Figure S3.** SEM images. (a-c) Pt@NGnP, (d-f) Pt@PGnP, (g-I) Pt@SbGnP at different magnifications: (a, d, g) ×50,000, scale bars = 5 µm; (b, e, h) ×25,000, scale bars = 2 µm; (c, f, i) ×10,000, scale bars = 1 µm.


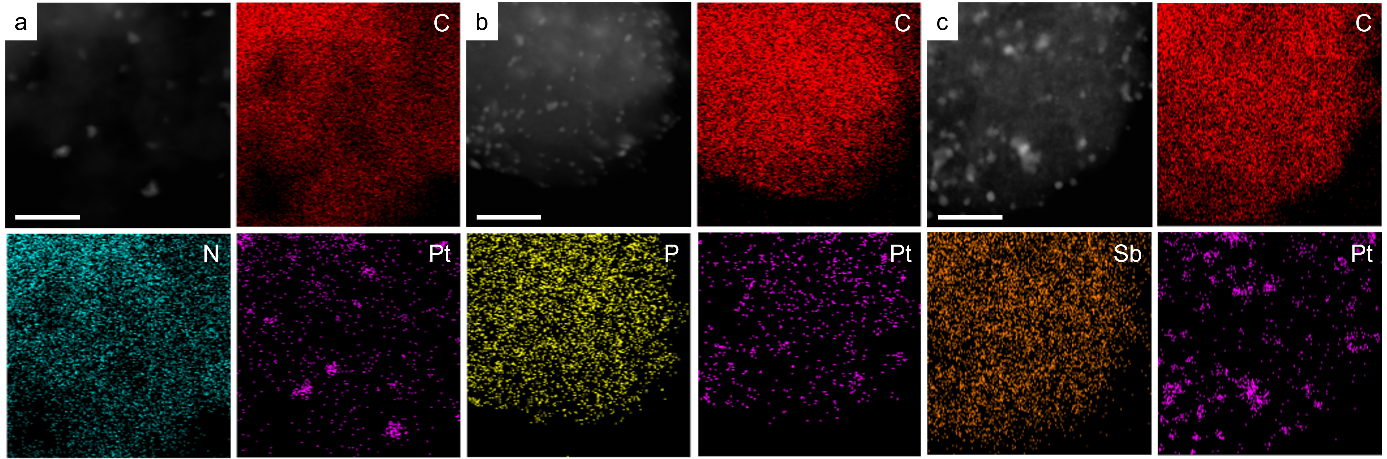


**Figure S4.** High−angle annular dark-field scanning transmission electron microscopy (HAADF−STEM) images and scanning transmission electron microscopy coupled energy−dispersive X-ray spectroscopy (STEM−EDS) element mappings. (a) Pt@NGnP; (b) Pt@PGnP; (c) Pt@SbGnP. Scale bars in (a-c): 25 nm.

**Figure S5.** XRD patterns of Pt@XGnPs (X = N, P, or Sb). Asterisks in Pt@XGnPs denote the Pt crystal lattice.

**Figure S6.** XPS survey spectra of Pt@XGnPs (X = N, P, or Sb).





**Figure S7.** The k spaces fitting curves: (a) Pt foil, (b) PtO_2_, (c) Pt@NGnP, (d) Pt@PGnP, (e) Pt@SbGnP.



**Figure S8.** The r spaces fitting curves: (a) Pt foil, (b) PtO_2_, (c) Pt@NGnP, (d) Pt@PGnP, (e) Pt@SbGnP.

**
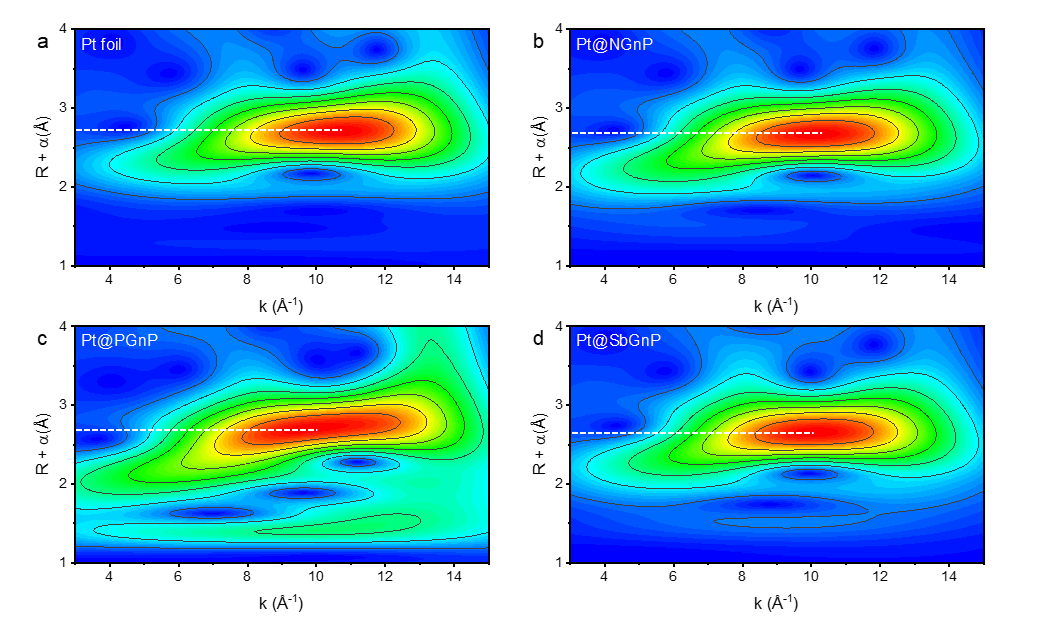
**

**Figure S9.** Wavelet transforms for the Pt L_3_-edge: (a) Pt foil; (b) Pt@NGnP; (c) Pt@PGnP; (d) Pt@SbGnP.


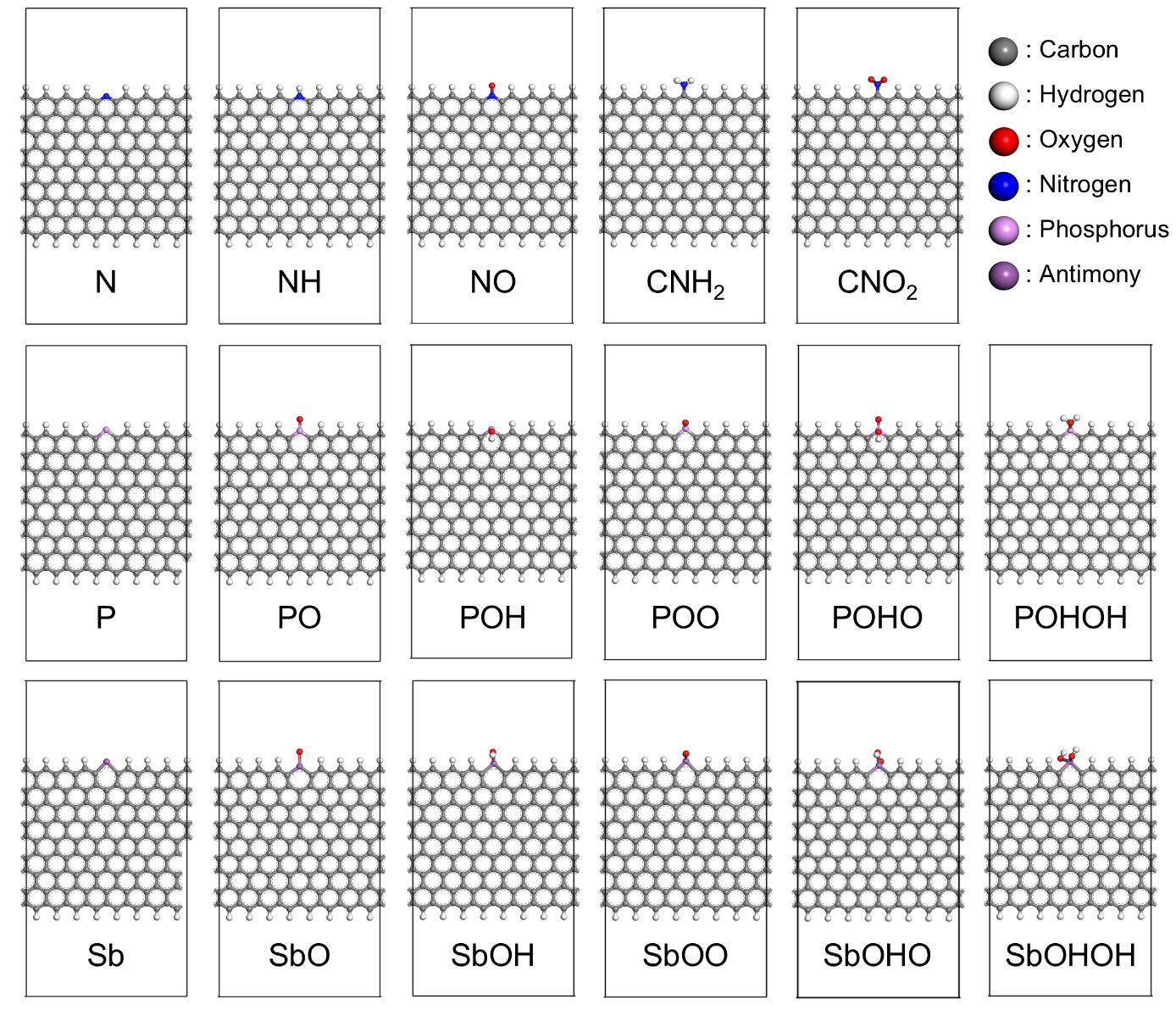


**Figure S10.** DFT optimized zigzag XGnPs (X = N, P, or Sb) models. Note that functional groups consisting of H and O were theoretically investigated for each dopant element.


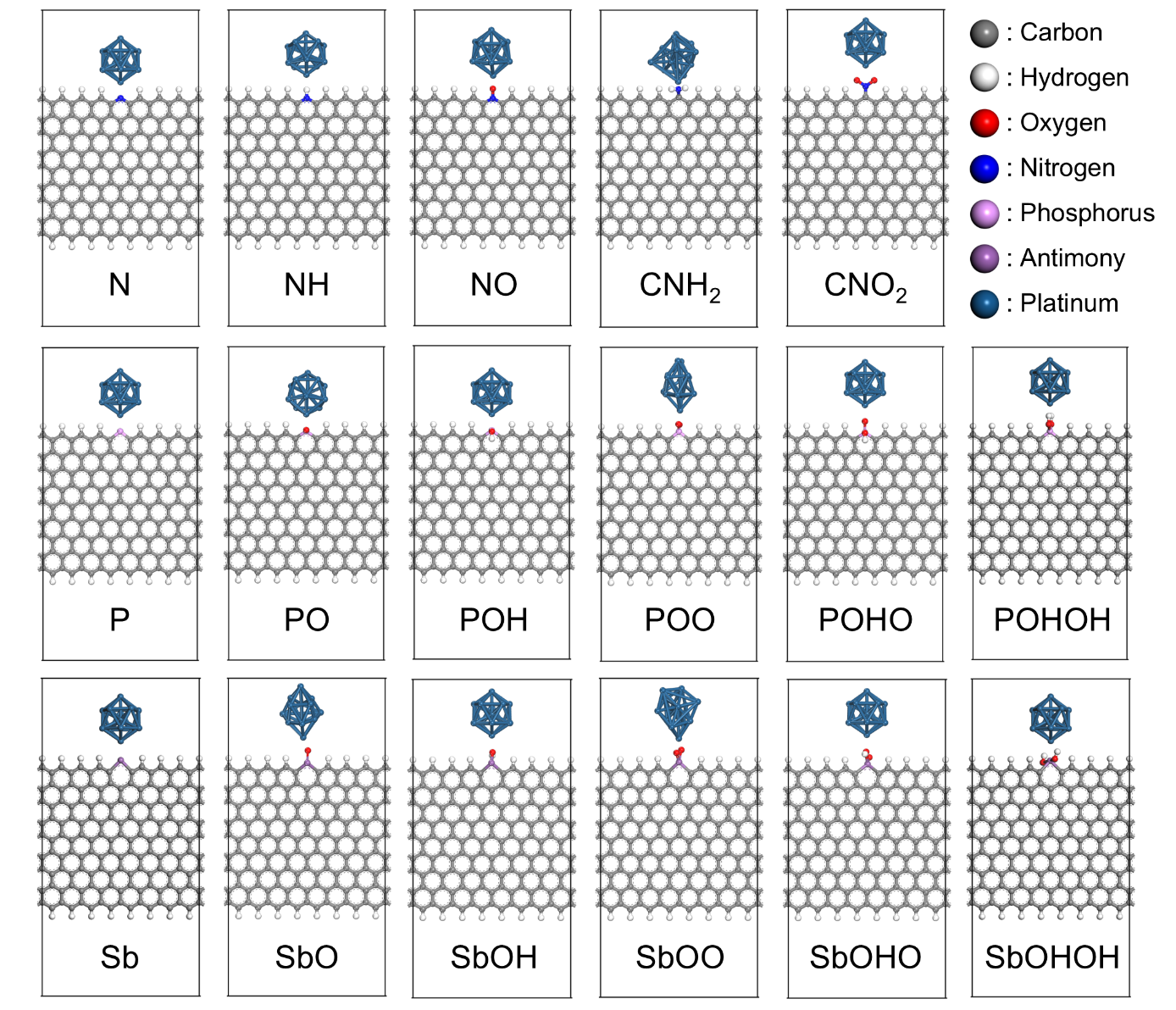


**Figure S11.** DFT optimized Pt@XGnPs (X = N, P, or Sb) models.


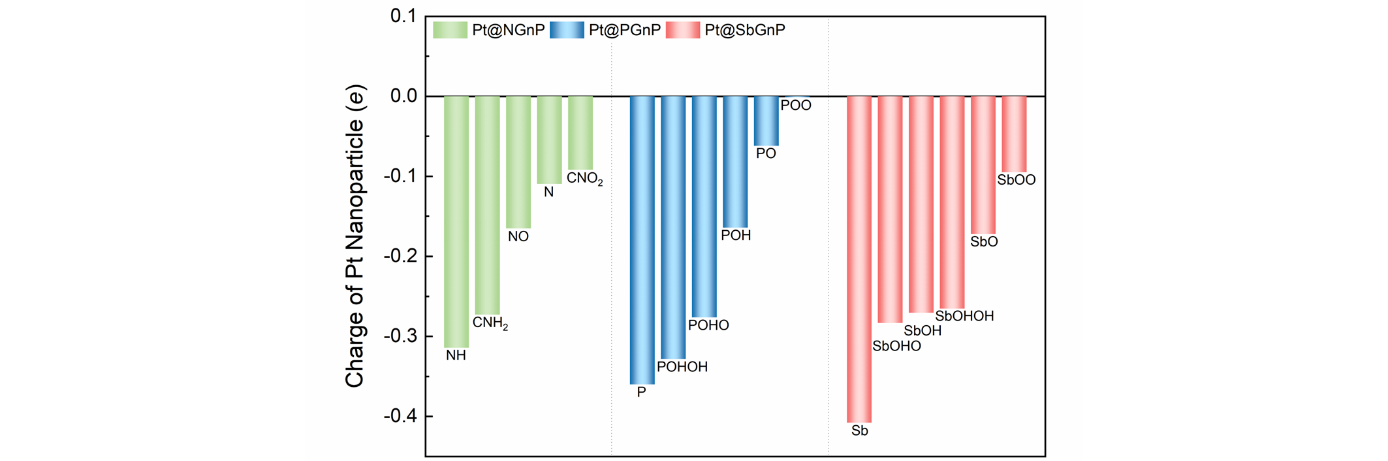


**Figure S12.** Calculated Pt NP charge (*e*) of Pt@XGnPs (X = N, P, or Sb) models in Figure S9. Note that negative value indicates that electrons are transferred from XGnP to Pt NP.


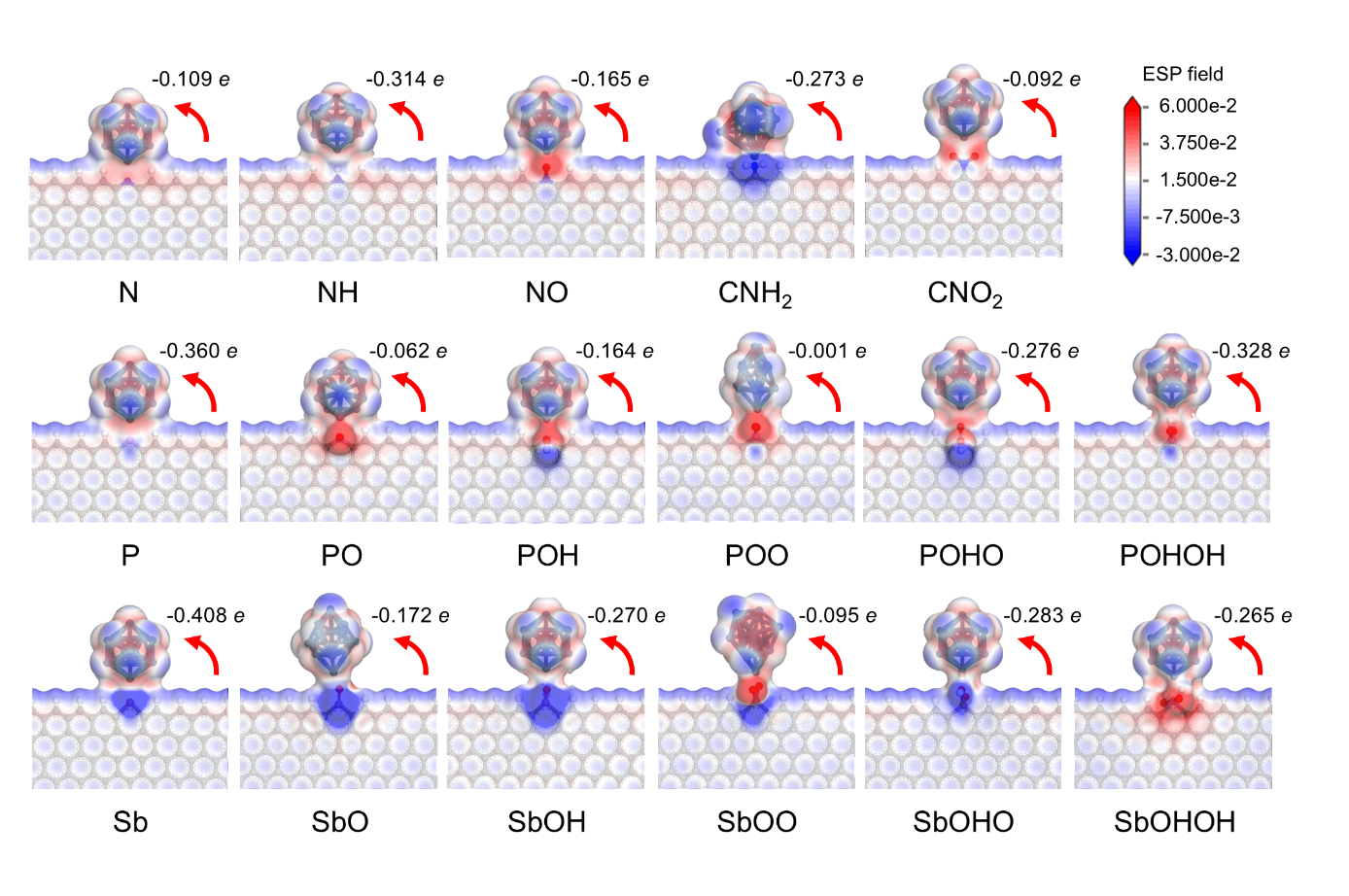


**Figure S13.** Electrostatic potential maps around the electron density isosurfaces of the optimized Pt@XGnPs (X = N, P, or Sb) models.



**Figure S14.** High-resolution C 1s XPS spectra. (a) Pt@NGnP; (b) Pt@PGnP; (c) Pt@SbGnP.





**Figure S15.** High-resolution XPS spectra. (a) P 2p XPS spectra of Pt@PGnP; (b) Sb 3d_3/2_ XPS spectra of Pt@SbGnP.





**Figure S16.** Raman spectra of Pt@NGnP, Pt@PGnP, and Pt@SbGnP.





**Figure S17.** HER polarization curves in 0.5 M aq. H_2_SO_4_. (a) NGnP and Pt@NGnP; (b) PGnP and Pt@PGnP; (c) SbGnP and Pt@SbGnP.



 **Figure S18.** Comparison graph of overpotentials at 10 mA cm^−2^ with the recently reported HER catalysts in 0.5 M aq. H_2_SO_4_.





**Figure S19.** (a) Electrochemical impedance spectroscopy (EIS) curves of the Pt/C and Pt@XGnP (X = N, P, or Sb) catalysts. (b) Histogram of the corresponding calculated charge transfer resistances (R_ct_).





**Figure S20.** Comparison of overpotentials at 10 mA cm^−2^ and exchange current densities. Spectroscopic characterizations of Pt/C, Pt@NGnP, Pt@PGnP and Pt@SbGnP.


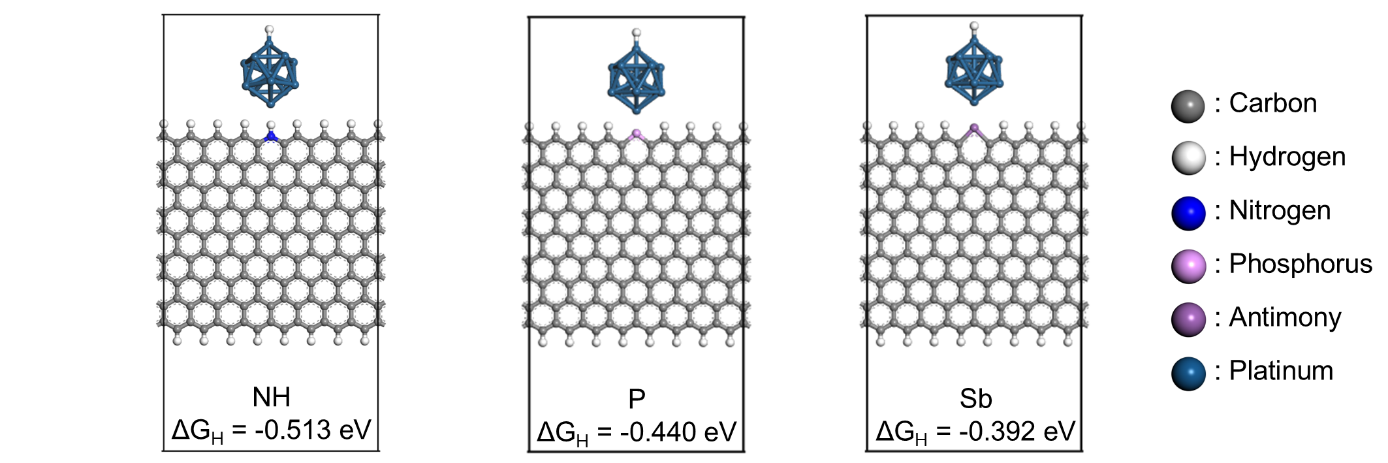


**Figure S21.** DFT optimized models of H-adsorbed Pt@XGnP (X = N, P, or Sb) structures and H-adsorption free energies.





**Figure S22.** (a) Copper UPD curves in 0.5 M aq. H_2_SO_4_ in the absence (I) and presence (II-XI) of 5 mM CuSO_4_ on Pt/C. For II-XI, the electrode was polarized at 0.22, 0.23, 0.24, 0.25, 0.26, 0.27, 0.28, 0.29, 0.30 and 0.31 V for 100 s, respectively, to form the UPD layer. Copper UPDs in 0.5 M aq. H_2_SO_4_ in the absence and presence of 5 mM CuSO_4_: (b) Pt/C; (c) Pt@NGnP; (d) Pt@PGnP; (e) Pt@SbGnP. The electrode was polarized at 0.31 V for 100 s to form the UPD layer.

Note: The underpotential deposition (UPD) of copper measurements were conducted in 0.5 M aq. H_2_SO_4_. Prior to Cu stripping in the absence and presence of 5 mM CuSO_4_ at a scan rate of 10 mV s^−1^, a UPD layer was formed by polarizing the electrode at 0.314 V for 100 s.





**Figure S23.** Comparison of the electrochemical surface areas and number of active sites using the Cu-UPD method.





**Figure S24**. CV curves measured at different scan rates from 10 to 100 mV s^−1^ in 0.5 M aq. H_2_SO_4_: (a) Pt/C; (b) Pt@NGnP; (c) Pt@PGnP; (d) Pt@SbGnP. (e) Capacitive current at middle potential of the CV curves as a function of the scan rates for Pt/C, Pt@NGnP, Pt@PGnP, and Pt@SbGnP.





**Figure S25**. TOF values of the Pt/C, Pt@NGnP, Pt@PGnP and Pt@SbGnP with other recently reported HER catalysts in 0.5 M aq. H_2_SO_4_.





**Figure S26.** Linear sweep voltammetry (LSV) curves measured before and after adding ^−^SCN ions to the 0.5 M aq. H_2_SO_4_ solution: (a) Pt@NGnP; (b) Pt@PGnP; (c) Pt@SbGnP. Chronoamperometry (CA) curves before and after the addition of ^−^SCN ions to the 0.5 M aq. H_2_SO_4_ solution: (d) Pt@NGnP; (e) Pt@PGnP; (f) Pt@SbGnP.





**Figure S27.** Comparison of overpotential changes of Pt/C and Pt@XGnPs before and after 10,000 cycles.





**Figure S28.** (a) Current-time curve of the Pt/C, Pt@NGnP, Pt@PGnP and Pt@SbGnP electrocatalysts conducted at 20 mV of overpotential for 40 h. (b) The losses in current densities between 0 h and after 40 h were compared.


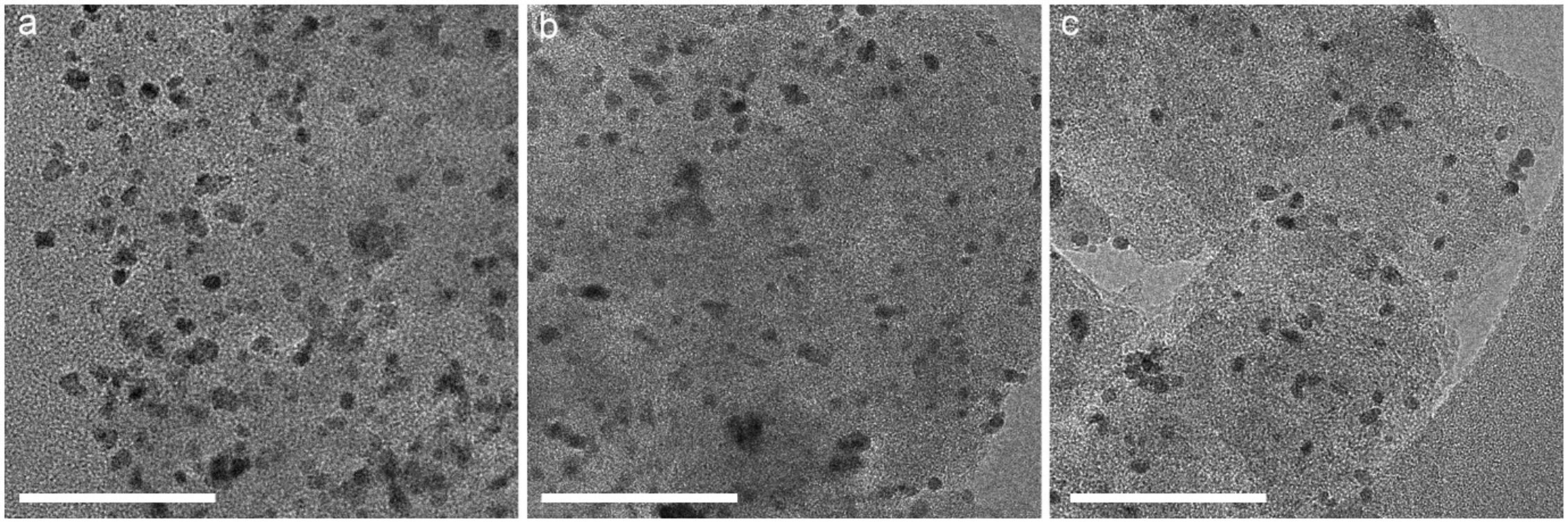


**Figure S29.** TEM images obtained after durability tests: (a) Pt@NGnP; (b) Pt@PGnP; (c) Pt@SbGnP. Scale bars in (a-c): 50 nm.


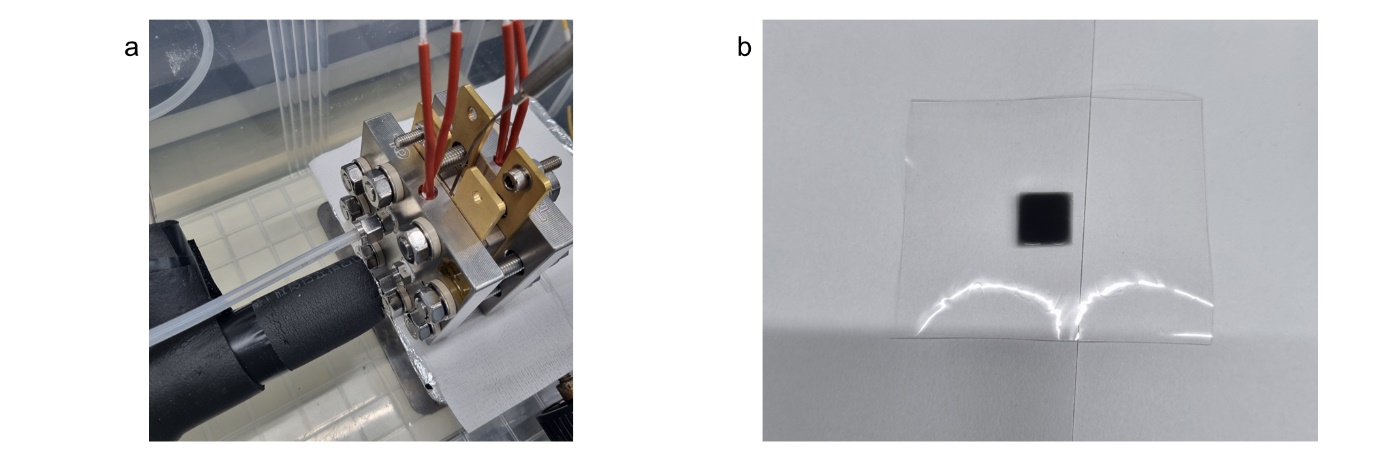


**Figure S30.** (a) Photograph of the actual PEMWE cell used in experiments. (b) Image of the MEA prepared using the electrospray technique.





**Figure S31.** XRD patterns of Pt@XGnP (X = N, P, or Sb) catalysts after durability tests.





**Figure S32.** High-resolution XPS spectra of the Pt@SbGnP catalyst after the durability test: (a) C 1s; (b) Sb 3d_3/2_; (c) Pt 4f





**Figure S33.** Current-potential curves of Pt wire in highly pure H_2_-saturated 0.5 M aq. H_2_SO_4_ solution, used to calibrate the Ag/AgCl electrode with respect to RHE. Scan rate: 1 mV s^−1^.

**Table S1.** Pt content in Pt@XGnPs (X = N, P, or Sb) from the ICP-OES results

| Catalysts | Pt content (wt. %) |
| --- | --- |
| Pt@NGnP | 12.29 |
| Pt@PGnP | 11.74 |
| Pt@SbGnP | 8.47 |

**Table S2.** EXAFS fitting parameters^a^ for the Pt L_3_-edge of Pt foil, PtO_2_, and Pt@XGnPs (X = N, P, or Sb)

| **Sample** | **Path** | **N^b^** | **R (Å)^c^** | **σ^2^×10^3^ (Å^2^)^d^** | **ΔE_0_ (eV)^e^** | **R factor** |
| --- | --- | --- | --- | --- | --- | --- |
| **Pt foil** | Pt-Pt | 12 | 2.77 | 4.82 | 7.99 | 0.010 |
| **PtO_2_** | Pt-O | 6 | 2.03 | 3.50 | 1.50 | 0.022 |
| **Pt@NGnP** | Pt-N | 3.9 | 2.61 | 6.95 | 3.69 | 0.005 |
|  | Pt-Pt | 9.9 | 2.74 | 4.72 |  |  |
| **Pt@PGnP** | Pt-O  Pt-P | 1  3 | 1.86  2.52 | 3.12  3.62 | 4.64 | 0.031 |
|  | Pt-Pt | 10.3 | 2.74 | 7.96 |  |  |
| **Pt@SbGnP** | Pt-O | 1.9 | 1.70 | 6.24 | 4.01 | 0.019 |
|  | Pt-Pt | 10.5 | 2.75 | 4.50 |  |  |

^a^ S_o_^2^ was fixed at 0.86.^[11]^ ^b^ *N*: coordination numbers; ^c^ *R*: bond distance; ^d^ *σ^2^*: Debye-Waller factors; ^e^ *ΔE_0_*: the inner potential correction. R factor: goodness of fit. according to the experimental EXAFS fit.

**Table S3.** Comparison of overpotential at 10 mA cm^−2^ and Tafel slopes of recently reported Pt nanoparticle based HER catalysts in 0.5 M aq. H_2_SO_4_

| Catalysts |  | η_10_  (mV) | Tafel slope (mV dec^−1^) | References |
| --- | --- | --- | --- | --- |
| **Pt@SbGnP** |  | **15.3** | **27.8** | **This work** |
| **Pt@PGnP** |  | **16.0** | **28.4** | **This work** |
| **Pt@NGnP** |  | **16.5** | **28.8** | **This work** |
| Pt-GT-1 |  | 18.0 | 24.0 | Nat Energy. **2018,** 3, 773. |
| Pt/Mxene |  | 34.0 | 29.7 | Adv. Funct. Mater. **2022,** 2110910. |
| TBA-Ti_3_C_2_T*_x_*-Pt-20 |  | 55.0 | 65.0 | ACS Sustain. Chem. Eng. **2019,** 7, 4266. |
| Pt@Ni ZIF-NC |  | 27.0 | 21.0 | J. Energy Chem. A. **2022,** 65, 48 |
| F-SnO_2_@Pt |  | 42.0 | 34.0 | ACS Nano **2022,** 16, 1625. |
| Pt-MoO_2_@PC |  | 20.0 | 22.0 | J. Energy Chem. A. **2020,** 8, 10409. |
| Pt Cs/MoO_2_ NSs-L |  | 47.0 | 36.0 | Nano Energy **2019,** 62, 127. |
| Pt-WO_3_ |  | 39 | 32.3 | Nano Energy **2020,** 71, 104653. |
| Pt/GNs |  | 25 | 33 | Carbon **2018,** 137, 405. |
| EG-Pt/CoP-1.5 |  | 21 | 42.5 | Energy Environ. Sci. **2019,** 12, 2298. |
| Pt-CQDs/Gr |  | 38 | 40 | J. Power Sources. **2020,** 451, 227770. |
| Pt/V_2_CT*x* |  | 67 | 20.6 | Appl. Surf. Sci. **2022,** 582, 152481. |
| siloxene-p-Pt |  | 23 | 25.7 | Appl. Catal. B. **2022,** 304, 121008. |
| 3% Pt-MoS_2_ |  | 67.4 | 76.2 | Nano Energy **2022,** 94, 106913. |
| Pt-25PG |  | 43 | 61 | Materials **2022,** 15, 73 |
| 10Pt@HN-BC |  | 47 | 35 | Int. J. Hydrog. Energy. **2018,** 43, 6167. |
| Pt-CNT |  | 41 | 49 | J. Energy Chem. **2020,** 51, 280. |
| Pt@GO@Ni-Cu@NF |  | 31 | 51 | Appl. Surf. Sci. **2020,**505, 144571. |
| Pt-CQDs/Gr-C400 |  | 38 | 40 | J. Power Sources. **2020,** 451, 227770. |
| Pt1.88-PVA |  | 34 | 31 | Ionics **2021,** 27, 4885. |
| Pt/Na_4_Ge_9_O_20_-PANI |  | 24 | 52 | Int. J. Hydrog. Energy. **2019,** 44, 31062. |
| Pt1.8MoS_2_ |  | 80 | 48 | ACS Appl. Mater. Interfaces. **2018,** 10, 8702. |
| Mo_2_C/CFP-Pt |  | 27 | 30 | J Catal. **2020,** 384, 169. |
| Mo_2_C@NC@Pt |  | 27 | 28 | ACS Appl. Mater. Interfaces. **2019,** 11, 4047. |
| HCS-N-Pt |  | 14.4 | 22 | ACS Appl. Mater. Interfaces. **2018,** 10, 43561. |
| Pt/G-MoS_2_ |  | 33 | 23 | Carbon **2018,** 139, 369. |
| Pt-CoO/p-CNF |  | 26 | 31.5 | J. Energy Chem. **2021,** 52, 33. |
| Pt/VC |  | 38 | 132.8 | Front. Mater. **2019,** 6, 251. |
| CPt@ZIF-67 |  | 50 | 27.1 | J. Energy Chem. A. **2019,** 7, 6543. |
| 3ZIF-67-Pt/RGO |  | 14.3 | 13.6 | ACS Appl. Mater. Interfaces. **2020,** 12, 10359. |
| S–M–5Pt |  | 17 | 78 | Adv. Funct. Mater. **2020,**30, 2000693. |
| Pt/rGO/GCE |  | 30 | 33 | J. Iran. Chem. Soc. **2019,** 16, 101. |
| Pt-CQDs/MWCNT |  | 35.3 | 34 | Chem. Eng. J. **2021,** 408, 127271. |
| PtNP/rGO-MWCNT |  | 11 | 28.6 | Chin Chem Lett. **2020,** 31, 1540-1544 |
| WC@C@Pt |  | 30 | 26 | Energy Stor. Mater. **2018,** 10, 268. |
| Pt-NCS-2 |  | 22 | 30 | Int. J. Hydrog. Energy. **2019,** 44, 31121. |
| Pt/Ti_0.9_Mo_0.1_O_2_ |  | 26 | 36 | Chin Chem Lett. **2021,** 32, 765. |
| CDs/Pt-PANI |  | 30 | 41.7 | Appl. Catal. B. **2019,** 257, 117905. |
| Pt@POMOF-1/KB |  | 23 | 71.29 | CrystEngComm. **2018,** 20, 5387. |
| Pt_c_/C |  | 37.5 | 30.9 | J. Energy Chem. A. **2021,** 9, 21972. |
| Pt–MoO_2_@PC |  | 20 | 22 | J. Mater. Chem. A. **2020,** 8, 10409. |
| Pt–TiO_2−_*_x_* NS |  | 35 | 32 | Nanoscale **2020,** 12, 11055. |
| Pt@PDG |  | 70 | 26.52 | New J. Chem. **2021,** 45, 21670. |
| Pt@CdSe-OCP |  | - | 61.3 | Chem. Mater. **2020,** 32, 2420. |
| Ni_3_[Fe(CN)_6_]_2_/Pt |  | 59 | 31 | Inorg. Chem. Front. **2018,** 5, 1683. |
| 80Pt/C-MOF |  | 42.1 | 24.5 | J. Mater. Chem. A. **2019,** 7, 20239. |
| Pt-PMo/ZIF-67-800 |  | 26 | 30 | Appl. Catal. B. **2021,** 298, 120579 |
| Pt/CNT-H |  | 19 | 60.3 | Mater. Lett. **2022,** 312, 131704 |
| PtCNP_2_ |  | 22 | 31.2 | J. Mater. Chem. A. **2022,** Advance Article |
| CTAs@Pt@NCBs |  | 27.42 | 37.5 | Chem. Eng. J. **2022,** 429, 132259. |
| MoS_2_–Pd–Pt |  | 64 | 64 | ACS Appl. Energy Mater. **2021,** 4, 10748. |
| Pt–TBA–Ti_3_C_2_T*_x_* |  | 67.8 | 69.8 | J Ind Eng Chem. **2020,** 59, 1822. |
| Bm-5d-Pt |  | 30 | 20 | Adv. Funct. Mater. **2021,**31, 2105372. |
| Pt@DNHC |  | 41 | 38 | RSC Adv. **2020,** 10, 930. |
| Pt–Ni_3_N/Ni@C |  | 45 | 47.3 | Electrochim. Acta. **2019,** 320, 134597. |
| Pt/CNTs-ECR |  | 34 | 26 | J. Mater. Chem. A. **2019,** 7, 15364. |
| HM-PtNPs |  | 33 | 33 | J Mater Sci Technol. **2020,** 46, 185. |
| Pt/PGaN |  | 98 | 85 | Chem. Phys. Lett. **2019,** 737, 136796. |
| MoC_1−_*_x_*/Pt-600-NPs |  | 30 | 31 | Adv. Sci. **2019,** 6, 1802135. |
| 0.8%Pt-Naf-CV |  | 33 | 33 | J. Colloid Interface Sci. **2020,** 566, 505. |

References

[1] I.-Y. Jeon, M. Choi, H.-J. Choi, S.-M. Jung, M.-J. Kim, J.-M. Seo, S.-Y. Bae, S. Yoo, G. Kim, H. Y. Jeong, N. Park, J.-B. Baek, *Nat. Commun.* **2015**, *6*, 7123.

[2] Á. Ganyecz, M. Kállay, *J. Phys. Chem. C* **2021**, *125*, 8551-8561.

[3] Z. Zhang, L. Yu, Y. Tu, R. Chen, L. Wu, J. Zhu, D. Deng, *Cell Rep.* **2020**, *1*, 100145.

[4] V. R. Jauja-Ccana, L. La-Torre-Riveros, A. Cordova-Huaman, G. Huayta, L. Manfredy, A. Naupa, M. Isaacs, A. La Rosa-Toro, *J. Electrochem. Soc.* **2023**, *170*, 040525.

[5] H. Liu, H. Wang, Y. Qian, J. Zhuang, L. Hu, Q. Chen, S. Zhou, *ACS Appl. Nano Mater.* **2019**, *2*, 7043-7050.

[6] A. Kumar, A. Ganguly, P. Papakonstantinou, *J. Phys. Condens. Matter.* **2012**, *24*, 235503.

[7] S. J. Clark, M. D. Segall, C. J. Pickard, P. J. Hasnip, M. I. J. Probert, K. Refson, M. C. Payne, *Z. fur Krist. - Cryst. Mater.* **2005**, *220*, 567-570.

[8] J. P. Perdew, K. Burke, M. Ernzerhof, *Phys. Rev. Lett.* **1996**, *77*, 3865-3868.

[9] A. Tkatchenko, M. Scheffler, *Phys. Rev. Lett.* **2009**, *102*, 073005.

[10] F. L. Hirshfeld, *Theor. Chim. Acta.* **1977**, *44*, 129-138.

[11] J. Cai, J. Chen, Y. Chen, J. Zhang, S. Zhang, *iScience* **2023**, *26*, 106730.
